# Identification and validation of Novel Estrogen Biosynthesis Biomarkers in Sinonasal Inverted Papilloma

**DOI:** 10.7150/ijms.101753

**Published:** 2025-01-01

**Authors:** Yi-Fang Yang, Sung-Huan Yu, Jia-Bin Liao, Yu-Hsuan Lin

**Affiliations:** 1 Department of Medical Education and Research, Kaohsiung Veterans General Hospital, Kaohsiung 813, Taiwan.; 2 Institute of Precision Medicine, College of Medicine, National Sun Yat-sen University, Kaohsiung 804, Taiwan.; 3 School of Medicine, College of Medicine, National Sun Yat-sen University, Kaohsiung 804, Taiwan.; 4 Department of Pathology and Laboratory Medicine, Kaohsiung Veterans General Hospital, Kaohsiung 813, Taiwan.; 5 Department of Otolaryngology, Head and Neck Surgery, Kaohsiung Veterans General Hospital, Kaohsiung 813, Taiwan.; 6 School of Medicine, National Yang Ming Chiao Tung University, Taipei 112, Taiwan.; 7 School of Medicine, Chung Shan Medical University, Taichung 402, Taiwan.; 8 School of Medicine, College of Medicine, National SunYat-sen University, Kaohsiung 804, Taiwan.

**Keywords:** Sinonasal inverted papilloma, Pathogenesis, Estrogen biosynthesis, Biomarker

## Abstract

**Background:** Sinonasal inverted papilloma (SNIP) is characterized by a high recurrence rate and potential for malignant transformation. Although metabolic reprogramming plays a role in benign neoplasms, the specific metabolic pathways and biomarkers involved in SNIP pathogenesis remain unclear.

**Methods:** RNA sequencing on paired SNIP and normal tissues identified altered genes with enzyme annotations and metabolic pathways by intersecting our cohort data (GSE270193, N=2) with the GSE193016 (N=4) dataset using Ingenuity Pathway Analysis. Functional and interaction assessments were performed using Metascape and STRING, with further validation via tissue microarray from an independent SNIP cohort (N=30).

**Results:** The estrogen biosynthesis pathway was significantly altered in both datasets. Five key biomarkers, AKR1B10, CYP1B1, CYP2C19, CYP3A5, and HSD17B13, were significantly altered in SNIP tissues. These markers, sharing Gene Ontology terms, showed significant correlations at both the transcript and protein levels. Functional analysis revealed enrichment in epithelial cell proliferation and regulation of EGFR signaling, suggesting a role in SNIP pathogenesis. Validation in an independent cohort confirmed elevated protein levels of these markers, all positively correlated with EGFR in SNIP tissues. Notably, AKR1B10, CYP2C19, and CYP3A5 exhibited specific expression patterns distinguishing SNIP from sinonasal squamous cell carcinoma.

**Conclusion:** Altered estrogen biosynthesis signaling plays a role in SNIP pathogenesis, revealing distinct biomarkers that could serve as novel diagnostic markers and therapeutic targets for SNIP management.

## Introduction

Sinonasal papilloma is the most common type of sinonasal tumor, with an incidence rate of 0.4-4.7% [1). The inverted variant is the most frequent subtype, known for its potential for local destruction, recurrence, and a propensity for malignant transformation 1-3. Extensive research has identified the primary oncogenic drivers in sinonasal inverted papilloma (SNIP) as mutations in the *EGFR* gene, particularly in exon 20 4-6 and infection by specific types of human papillomavirus (HPV) 7,8, which are essentially mutually exclusive 5,7. This underscores the crucial role of EGFR signaling in SNIP, distinguishing it biologically from other sinonasal papilloma variants 4. Factors contributing to SNIP recurrence include the activation of EGFR signaling 5, positive HPV status 9, and the presence of dysplasia 10. Squamous cell carcinoma (SCC) arising from SNIP often exhibits more *EGFR* mutations, but fewer high-risk HPV strains compared to *de novo* SCC 5,7. Despite these significant advancements, a comprehensive understanding of the pathogenesis of SNIP remains elusive.

Tumor cells possess a remarkable capacity for adaptation by exploiting various metabolic pathways to survive the hostile tumor microenvironment 11. This metabolic alteration is also observed in benign tumors to maintain their tumorigenic properties 12-14. For instance, research has indicated that metabolic changes are already present at the adenoma stage, marked by aberrant MYC expression, which activates 215 metabolic pathways to reprogram tumor cell metabolism and promote cell growth 12. In high-risk pituitary adenomas, characterized by elevated glucose transporter 1 (GLUT-1) levels, activated glucose metabolism may promote histone acetylation, leading to telomerase reverse transcriptase (TERT) upregulation to promote cell proliferation in pituitary tumors 13. Similarly, aldosterone-producing adenomas exhibit shifts in fatty acid β-oxidation and glycolysis, where an increase in lipid metabolism mediated by PPARα could affect lipid peroxidation, a hallmark of regulated cell death by ferroptosis, to support adrenocortical cell growth 14. Additionally, the study reveals an immunosuppressive tumor microenvironment with reduced CD45+ immune cell infiltration, suggesting metabolic adaptations provide survival advantages for aldosterone-producing adenomas. However, the extent to which alterations in metabolic pathways influence SNIP pathogenesis remains unclear.

Through bioinformatics analysis of RNA-sequencing databases, our study fills a crucial gap by identifying the dysregulation of the estrogen biosynthesis pathway in SNIP tissues. We demonstrated significant changes in AKR1B10, CYP1B1, CYP2C19, CYP3A5, and HSD17B13 expression between SNIP and control tissues, with these alterations observed at both transcript and protein levels. These biomarkers shared common Gene Oncology (GO) terms, and molecular interaction analyses revealed significant correlations between them. Further functional analysis of the overlapping genes correlated with each marker revealed enrichment in epithelial cell proliferation and positive regulation of EGFR signaling pathway, suggesting their collective role in SNIP pathogenesis. These findings lay the groundwork for developing diagnostic and therapeutic strategies based on these biomarkers and their associated pathways.

## Materials and Methods

### Library preparation and sequencing of SNIP patients

Tissue specimens from SNIP and paired normal tissues were collected from two patients who provided informed consent. The study was approved by KSVGH21-CT12-21. Using the TruSeq Stranded mRNA Library Prep Kit (Illumina, San Diego, CA, USA), the RNA was purified and prepared for sequencing per the manufacturer's instructions. In summary, mRNA was separated at high temperatures from 1 μg of total RNA using oligo(dT)-coupled magnetic beads. The first-strand complementary DNA (cDNA) was synthesized using random primers and reverse transcription. After double-strand cDNA synthesis, 3' adenylation, and adaptor ligation, the libraries were purified using the Beckman Coulter AMPure XP system (Beverly, USA). Library quality was evaluated using real-time PCR with an Agilent Bioanalyzer 2100. Sequencing was performed with 150 bp paired-end reads on the Illumina NovaSeq 6000 platform. RNA sequencing data were deposited in the Gene Expression Omnibus database (GEO) under the accession number GSE270193.

### Pathway identification and biomarker selection

The GSE193016 dataset, comprising mRNA microarray data from four SNIP samples and four normal tissue controls from patients with nasal septum deviation, was downloaded from GEO (https://www.ncbi.nlm.nih.gov/geo/). The dataset GSE270193 was normalized using DESeq2 (version 1.28.0), and genes showing significant changes were identified. For GSE193016, genes exhibiting a fold-change of 2.0 or greater were identified using GEO2R. Ingenuity Pathway Analysis (IPA) was used for identifying genes with enzyme annotation and significantly altered metabolic pathways shared between the datasets, focusing on those with a high percentage of overlapping genes and a p-value less than 0.05. Potential biomarkers were selected based on the shared significantly altered genes with enzyme annotations from both datasets.

### Quantitative reverse transcription real-time PCR (RT-qPCR)

TRIzol® Reagent (#15596018, Invitrogen, ThermoFisher Scientific, Waltham, MA, U.S.A.) was used to isolate total RNA from tissues per the manufacturer's instructions. A PrimeScript RT Reagent Kit (#RR037A, Takara Bio, Shiga, Japan) was used to synthesize cDNA. Real-time PCR was performed using qPCR BIO SyGreen Mix Lo-ROX SYBRTM Green PCR Master Mix (#PB20.11-01, PCR BIOSYSTEMS, U.S.A) to evaluate target gene expression. The primers used included: *AKR1B10*-F: GCAACGTTCTTGGATGCCTG; *AKR1B10*-R: TGGCTGAAATTGGAGACCCC; CYP1B1-F: GACTCGAGTGCAGGCAGAAT; *CYP1B1*-R: CAGGACATAGGGCAGGTTGG; *CYP3A5-F*: CTCCTCTATCTATATGGGACCCG; *CYP3A5*-R: AACAAAGGCAGAGGTGTGGG; *CYP2C19*-F: AGGATTGTAAGCACCCCCTG; *CYP2C19*-R: AAGTAATTTGTTATGGGTTCCCG; *HSD17B13-*F: ATCATGGCCACATCGTCACA; *HSD17B13*-R: CTCTGTGAAAGCCAACAGCG; *ACTB*-F: AGAAAATCTGGCACCACACC; *ACTB*-R: AGAGGCGTACAGGGATAGCA.

### Functional characterization and molecular interaction analyses

Functional annotation of the significantly altered genes was conducted using the Gene Ontology (GO) category database (https://geneontology.org). To assess the correlations between these biomarkers, we first extracted protein-coding genes from the two GEO datasets. For genes in the GSE193016 dataset that consisted of two or more probes, the expression levels were calculated as the average of these probes. We then identified the genes correlated with each biomarker across the 12 specimens in the two datasets and determined the intersection of these correlated genes, setting a threshold correlation coefficient of 0.7 for inclusion. Functional enrichment analysis of overlapping correlated genes was performed using Metascape (http://metascape.org). Protein-protein interactions of the biomarkers were explored using STRING (https://string-db.org), and these interactions were visualized using Cytoscape (https://cytoscape.org).

### SNIP tissue microarrays and immunocytochemistry analysis

Tissue microarray (TMA) slides containing SNIP, sinonasal malignancy, and control tissue samples were procured from SuperBioChips Laboratories (NH1001a; Seoul, Republic of Korea). For immunocytochemistry (ICC), a fluorescent multiplex staining kit (BioTnA, TATS01F, Kaohsiung, Taiwan) and specific antibodies targeting the proteins of interest were used. The procedure began with the fixation of tissue samples using 4% paraformaldehyde, followed by permeabilization with 0.2% Triton X-100. To block non-specific binding sites, 5% bovine serum albumin (BSA) was used. Primary antibodies were incubated overnight at 4 °C, followed by incubation with secondary antibodies at room temperature for 30 min. The stained slides were analyzed at the Li-Tzung Pathology Laboratory (Kaohsiung, Taiwan) using a BX61VS fully motorized fluorescence microscope (Olympus, Tokyo, Japan). CYP2C19 (1:200; K109611P, Solarbio), AKR1B10 (1:100; K109534P, Solarbio), HSD17B13 (1:100; A6256, Abclonal), CYP3A5 (1:200; BML-CR3350-0025, Enzo), and CYP1B1 (1:100; 18505-1-AP, Proteintech). A pathologist confirmed the quality of TMA. The ICC for each biomarker was quantified using the ImageJ software.

### Statistical analysis

Data are represented as mean ± standard deviation. Comparisons between groups were performed using the Mann-Whitney *U* test and Fisher's exact test. Tukey's post-hoc test was used for the RT-qPCR data. Correlation analyses were performed using Spearman correlation coefficients. All statistical tests were two-tailed, with significance set at p-values < 0.05.

## Results

### Estrogen biosynthesis pathway is upregulated in SNIP tissues

**Figure 1:**
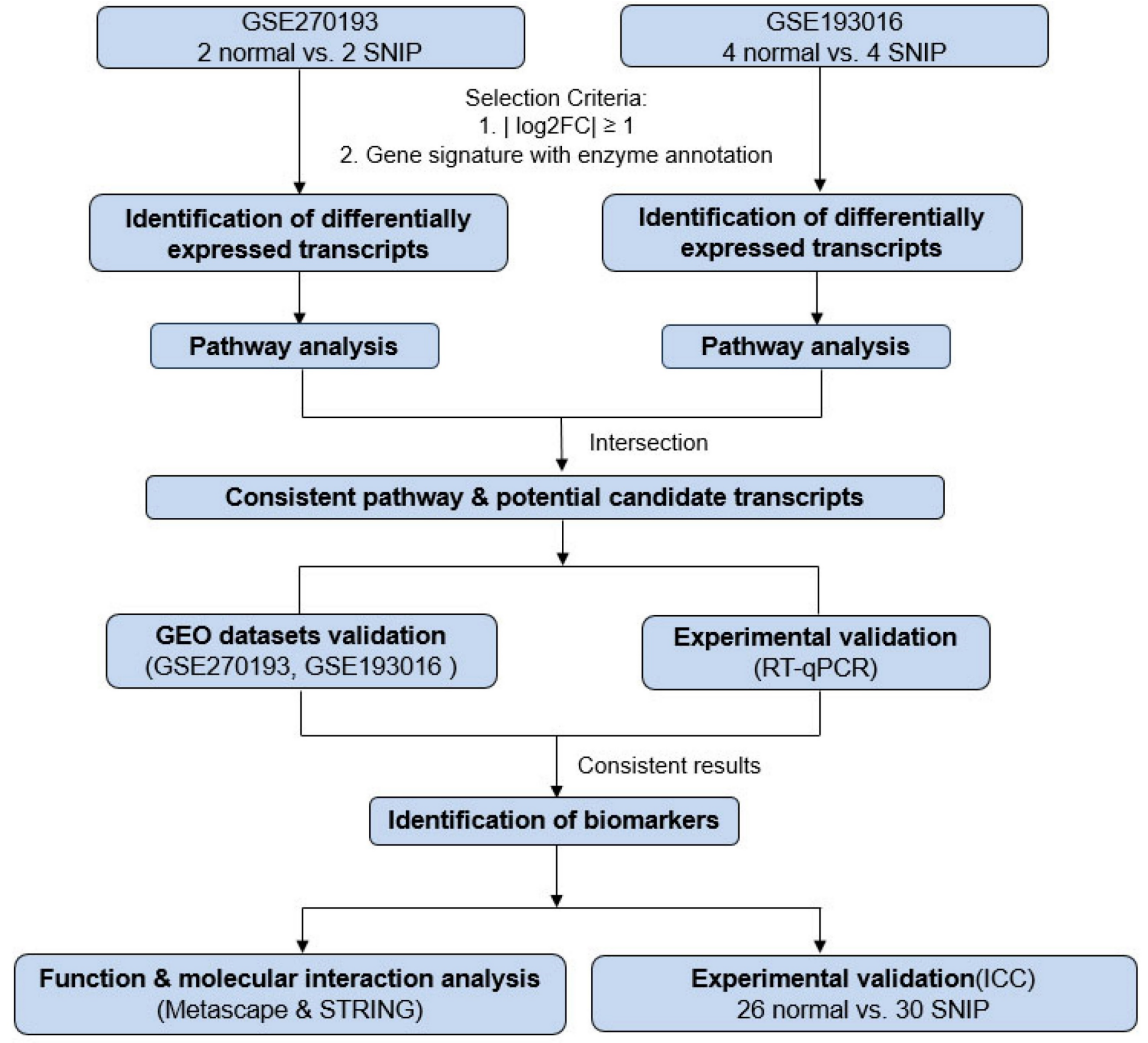
Schematic workflow for the identification and verification of biomarkers for sinonasal inverted papilloma (SNIP).

**Figure 2:**
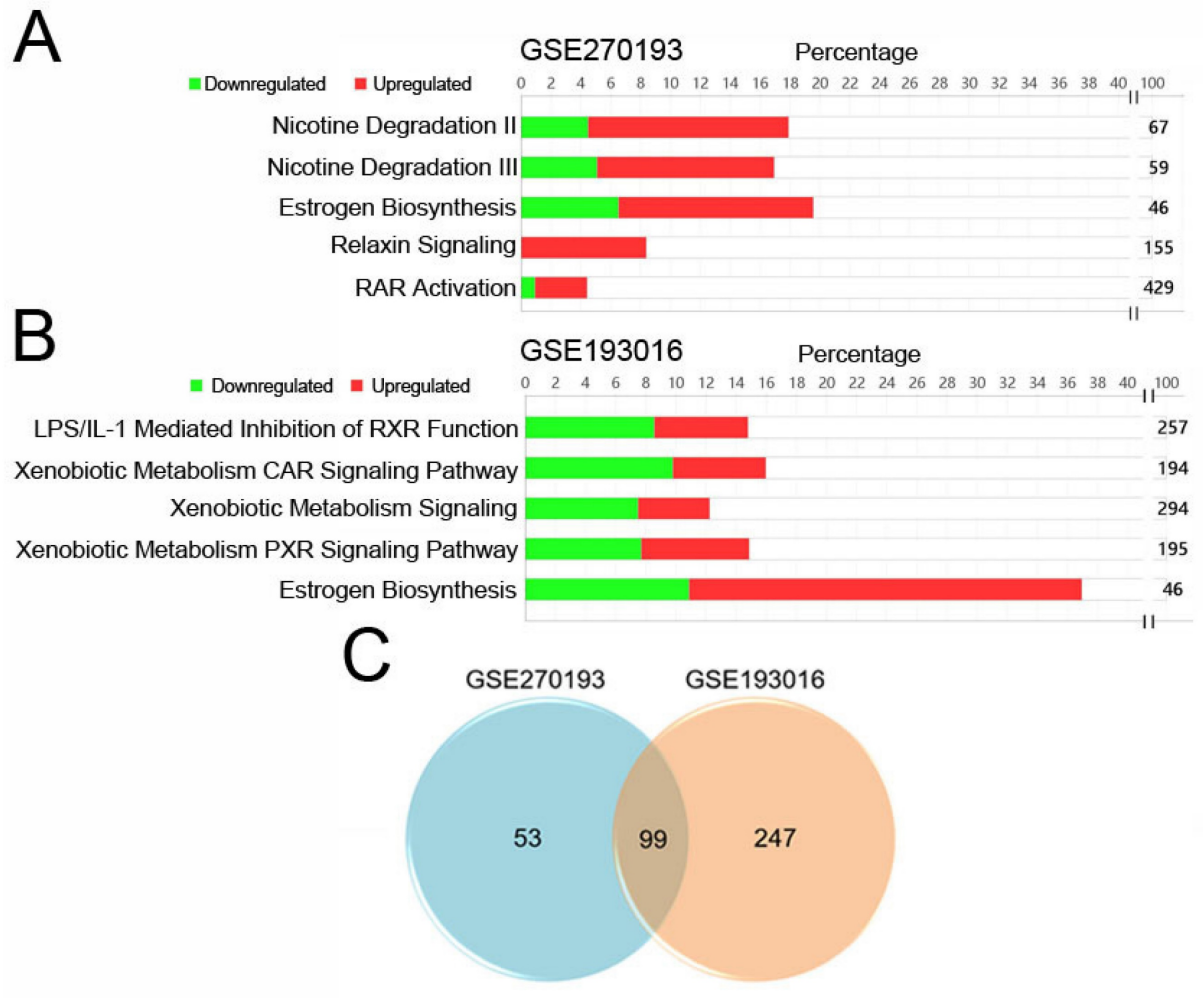
Identified estrogen biosynthesis pathways and candidate genes for SNIP. IPA pathway analysis of significantly changed genes with enzyme annotations for dataset (A) GSE270193 and (B) GSE193016. (C) Venn diagram showing overlap genes in both datasets.

The selection process for these datasets is illustrated in Figure 1. Using a threshold of |log2 fold change (log2FC) | ≥ 1, we identified 1740 and 2875 genes with significant changes in the GSE270193 dataset (2 paired control vs. 2 SNIP) and GSE193016 dataset (4 control vs. 4 SNIP), respectively. Subsequent canonical pathway analyses of significantly altered transcripts annotated with enzyme functions were performed using IPA. This analysis highlighted the top 5 significant pathways for each dataset, as shown in Figures 2A and 2B and detailed in Table S1 and Table S2. The estrogen biosynthesis pathway was the only common pathway identified in both datasets.

### *AKR1B10, CYP2C19,* and *CYP3A5* mRNA were upregulated in SNIP tissues

To identify genes with enzyme signatures that were consistently altered in our patients (Supplementary Material 1) and the GSE193016 dataset (Supplementary Material 2), we compared the results, which yielded 99 candidate transcripts (Figure 2C, Table S3). Among these, seven genes, *Aldo-Keto Reductase Family 1 Member B10* (*AKR1B10*), *Cytochrome P450 Family 1 Subfamily B Member 1* (*CYP1B1*), *Cytochrome P450 enzyme 2C19* (*CYP2C19*), *Cytochrome P450 Family 3 Subfamily A Member 5* (*CYP3A5*), *Hydroxysteroid 17-Beta Dehydrogenase 13* (*HSD17B13*), *Hydroxysteroid 17-Beta Dehydrogenase 2* (*HSD17B2*), and *Hydroxysteroid 17-Beta Dehydrogenase 6* (*HSD17B6*), were associated with the estrogen biosynthesis pathway according to the IPA gene set.

**Figure 3:**
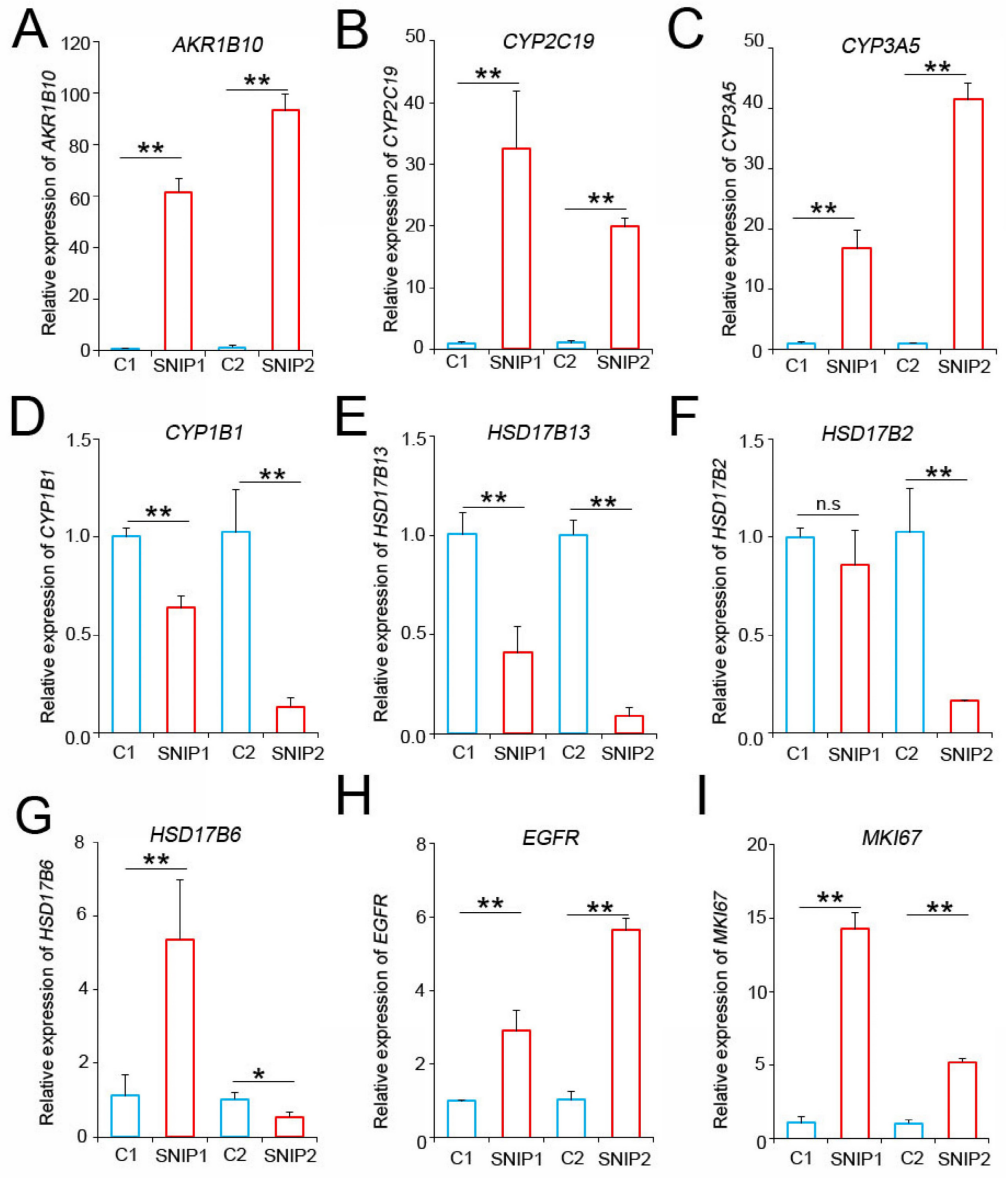
*AKR1B10*, *CYP2C19*, and *CYP3A5* mRNA levels were upregulated in SNIP tissues. RT-PCR analysis of (A) AKR1B10, (B) CYP2C19, (C) CYP3A5, (D) CYP1B1, (E) HSD17B13, (F) HSD17B2, (G) HSD17B6, (H) EGFR, and (I) MKI67 expression in SNIP versus paired normal tissues. Tukey's post hoc test was used to determine the statistical significance. *p < 0.05; **p < 0.01. n.s, not significant.

To validate these findings, we performed RT-qPCR analysis to assess the expression levels of these candidate biomarkers in tumor and normal tissues from our patients. The results indicated that the *AKR1B10*, *CYP2C19*, and *CYP3A5* levels were consistently and significantly upregulated in SNIP tissues compared to those in their normal counterparts (Figure 3A-C). Conversely, *CYP1B1* and *HSD17B13* were downregulated in SNIP tissues, which was consistent with the GSE193016 dataset (Figure 3D-E). The *HSD17B2* and *HSD17B6* expression levels were downregulated in the GSE193016 dataset; however, *HSD17B2* demonstrated no significant change in one patient and *HSD17B6* exhibited a paradoxical trend between the two patients in our cohort (Figure 3F-G). As a reference for SNIP, the *EGFR* and* MKI67* levels were significantly higher in tumor tissues than in paired normal tissues (Figure 3H-I).

### Expression levels of estrogen biosynthesis enzymes are associated with cell proliferation function in SNIP

To evaluate the potential contributions of these biomarkers to the pathogenesis of SNIP via estrogen biosynthesis, we searched for the functional annotations of the five biomarkers using the GO database. As shown in Supplementary Material 3, all biomarkers shared common GO terms across the biological process (BP), molecular function (MF), and cellular component (CC) categories. In the BP category, CYP1B1, CYP3A5, CYP2C19, and AKR1B10 shared multiple GO terms related to metabolic processes. Although HSD17B13 shares fewer GO terms with other genes, its associated terms, including “regulation of lipid biosynthetic process (GO:0046890)” and “regulation of lipid metabolic process (GO:0019216)”, suggest its involvement in sex hormone metabolism.

**Figure 4:**
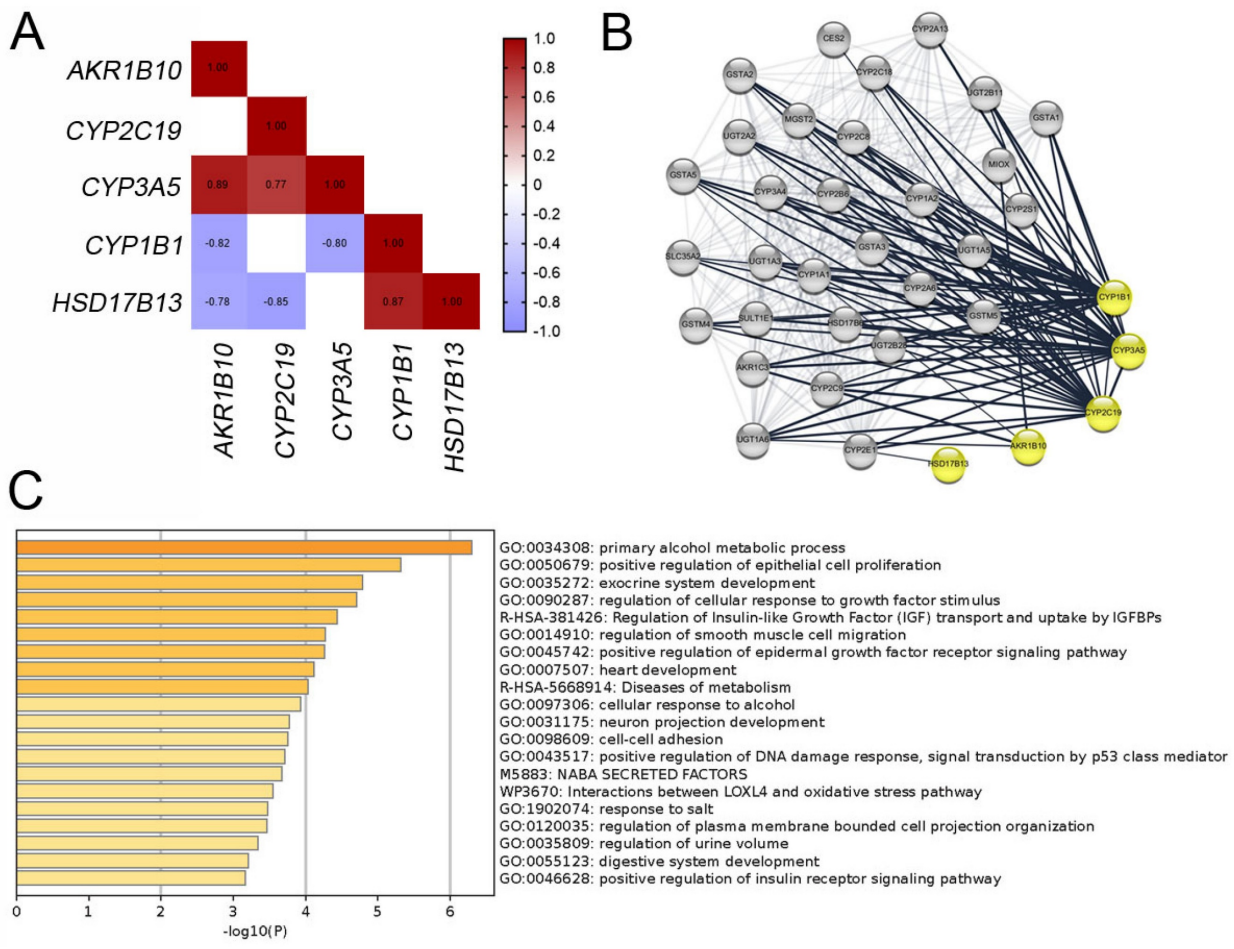
Correlation and functional enrichment analyses for biomarkers. (A) Correlations between mRNA levels among the biomarkers. (B) Protein interaction network for biomarkers visualized using Cytoscape. (C) Enriched terms for shared correlated genes of the biomarkers.

To assess the correlations among these biomarkers, we identified the correlated genes for each biomarker with a correlation threshold of 0.7 across 12 specimens from both datasets. Figure 4A shows Spearman's correlation coefficients among the biomarkers, indicating significant associations between most biomarkers. Further evaluation of the interactions between these five biomarkers using the STRING database (Figure 4B) suggested that these biomarkers may collaboratively contribute to SNIP development. To confirm the involvement of these biomarkers in SNIP tumorigenesis, we intersected the genes correlated with each biomarker. Subsequent functional enrichment analysis of these correlated genes using Metascape identified significant association with “positive regulation of epithelial cell proliferation (GO: 0050679)”, “regulation of cellular response to growth factor stimulus (GO: 0090287)”, and “positive regulation of epidermal growth factor receptor signaling pathway (GO: 0045742)” (Figure 4C).

### AKR1B10, CYP1B1, CYP2C19, CYP3A5, and HSD17B13 protein levels are upregulated in SNIP tissues

**Figure 5:**
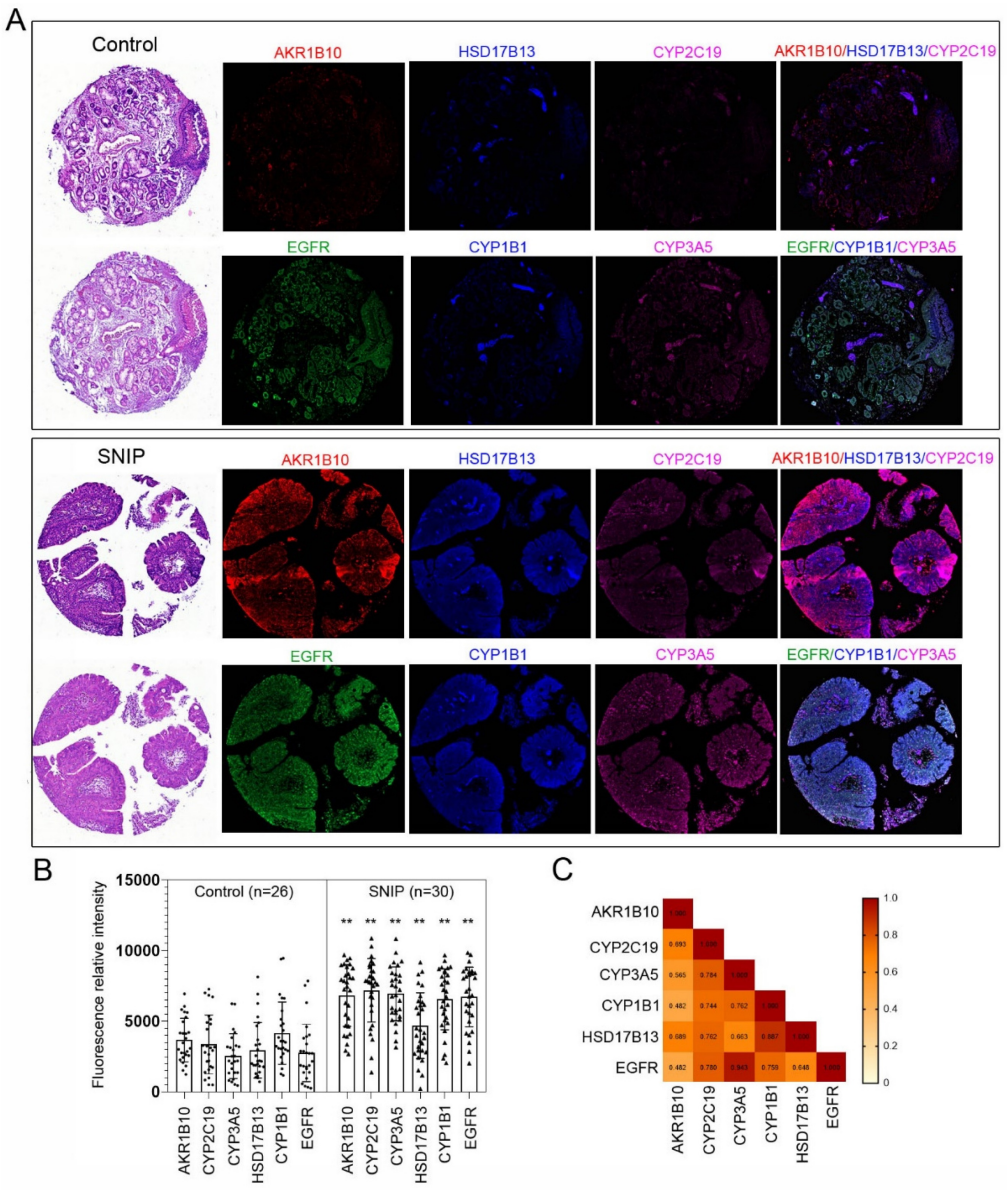
Estrogen biosynthesis enzymes were upregulated in SNIP samples. (A) Representative tissue section images of Immunocytochemistry for AKR1B10, HSD17B13, CYP2C19, CYP1B1, and CYP3A5 and EGFR expression in SNIP and control tissues. (B) Relative fluorescence intensity of biomarkers and EGFR in SNIP and control tissues. Mann-Whitney *U* test was used to determine the significance. **p < 0.01. (C) Correlation matrix among the AKR1B10, HSD17B13, CYP2C19, CYP1B1, CYP3A5 and, EGFR in SNIP tissues.

To validate our findings, immunocytochemistry was performed to assess the concentrations of these markers in tissue microarrays (TMAs; NH1001a) from another independent cohort (Table S4). The TMA included samples from 30 (34.9%) SNIP cases, 26 (30.2%) controls, and 30 (34.9%) squamous cell carcinomas (SCC) originating from the sinonasal cavities, with an average patient age of 50.20 ± 14.86 years, of which 72 (83.7%) specimens were from male patients. The five biomarkers consistently exhibited significantly higher expression levels in SNIP tissues than that in the controls (p < 0.01, Figure 5A-B). The p-values for AKR1B10, CYP2C19, and CYP3A5 were all less than 0.00001. Additionally, there was a significant positive correlation between each pair of biomarkers, and EGFR was positively and significantly correlated with all the biomarkers (Figure 5C). This corresponded with the results of the functional enrichment analysis of the correlated genes, which identified an association with the regulation of the epidermal growth factor receptor signaling pathway (Figure 4C). Further analyses indicated that there were no significant differences in age or sex between the high- and low-expression groups for the biomarkers (categorized by the mean value of each biomarker expression) in the SNIP tissues. However, EGFR expression levels were significantly higher in the subgroup with elevated HSD17B13, CYP2C19, CYP1B1, and CYP3A5 expression than that in the low-expression subgroup (Table 1).

### Biomarkers expression are downregulated in malignant tissues

**Figure 6:**
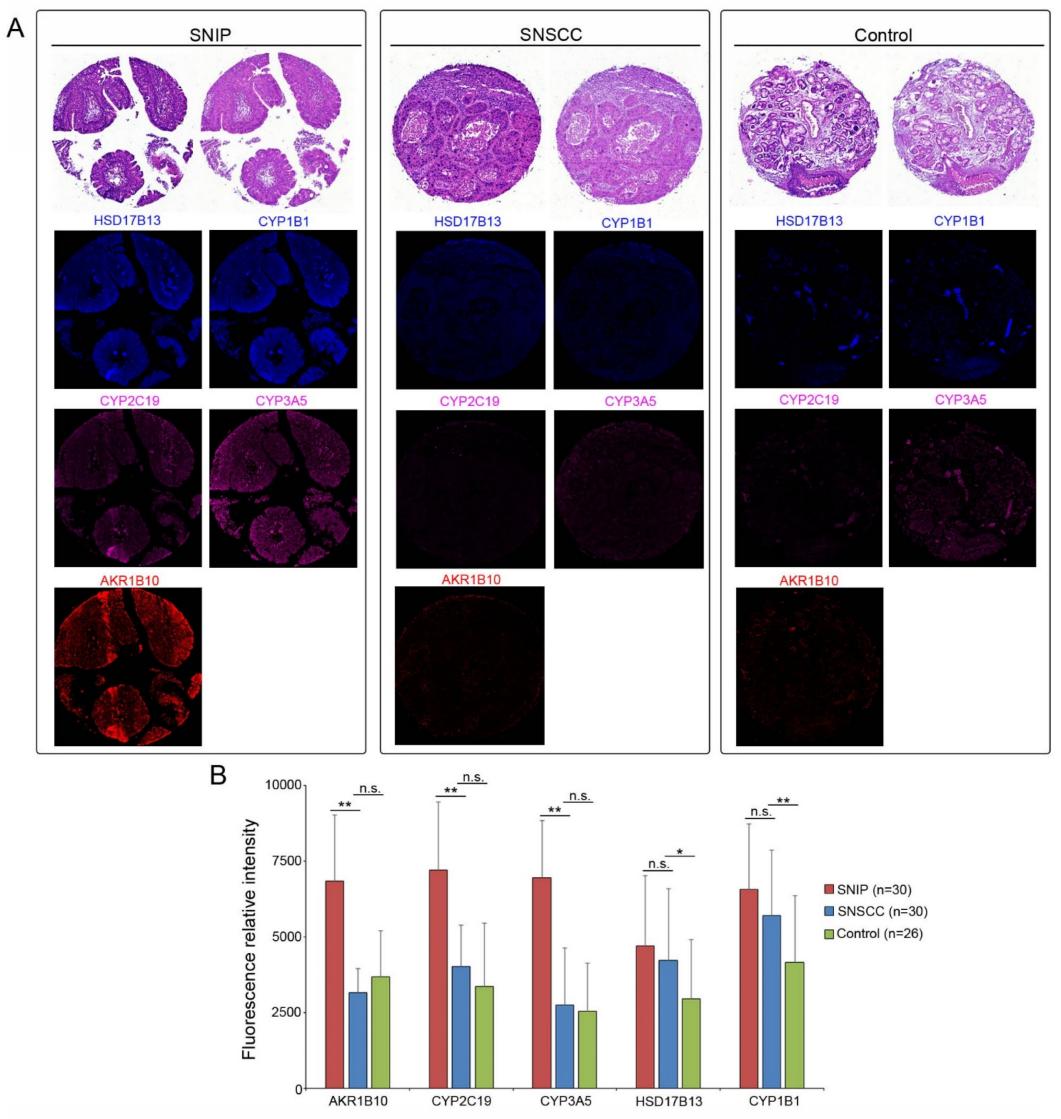
AKR1B10, CYP2C19, and CYP3A5 protein levels were specific to SNIP. Representative immunocytochemistry images of (A) SNIP tissue, sinonasal squamous cell carcinoma (SNSCC) tissue, and control tissue. (B) Relative fluorescence intensity of biomarkers in SNIP, SNSCC, and control tissues. Mann-Whitney *U* test was used to determine the significance. *p < 0.05; **p < 0.01. n.s, not significant.

Given that these estrogen biosynthesis-related biomarkers are implicated in the tumorigenesis of various cancer subtypes 15-17, we assessed their expression levels in sinonasal squamous cell carcinoma (SNSCC). As shown in Figure 6A, ICC revealed that the protein expression of AKR1B10 (p < 0.01), CYP2C19 (p < 0.01), and CYP3A5 (p < 0.01) was significantly downregulated in SNSCCs compared to that in SNIP tissues (Figure 6B). However, no significant differences were observed for HSD17B13 (p = 0.5687) or CYP1B1 (p = 0.1285). Further comparison of the expression levels of these three biomarkers in SNSCCs and controls demonstrated no significant differences in AKR1B10 (p = 0.3681), CYP2C19 (p = 0.1074), and CYP3A5 (p = 0.8103) (Figure 6A-B), suggesting that AKR1B10, CYP2C19, and CYP3A5 may be specific to SNIP rather than SNSCC.

## Discussion

Our research elucidated the significant differential expression of key markers involved in the estrogen biosynthesis pathway, AKR1B10, CYP1B1, CYP2C19, CYP3A5, and HSD17B13, with notable variations observed at the transcript level in SNIP tissues compared to normal tissues across the two cohorts. Comprehensive molecular interaction analyses revealed significant correlations between these biomarkers, and functional enrichment analysis of the genes correlated with these markers revealed associations with epithelial cell proliferation and positive regulation of EGFR signaling pathway. These findings suggest that a coordinated regulatory mechanism contributes to SNIP pathogenesis. Further validation in an independent cohort confirmed that these five biomarkers were significantly overexpressed in patients with SNIP compared with controls. Additionally, they exhibited significant correlations with EGFR, further supporting their potential role in SNIP pathogenesis through involvement in EGFR signaling. Specifically, AKR1B10, CYP2C19, and CYP3A5 showed distinct expression patterns in SNIP, distinguishing them from sinonasal malignancies and thereby underscoring their potential specificity for SNIP.

Estrogen synthesis is divided into gonadal, which releases estrogen into the bloodstream, and extra-gonadal, which acts locally at the synthesis site 18. Estrogen signaling is primarily mediated by estrogen receptors (ERs), which are involved in genomic effects through the nuclear-initiated pathway that triggers transcriptional changes in estrogen-responsive genes and rapid non-genomic activities via the membrane-initiated pathway 19,20. The latter pathway activates ERs to interact with other membrane receptors or trigger signaling cascades such as the MAPK/ERK, PI3K/AKT, and tyrosine cascades. ERs can also initiate gene transcription in a ligand-independent manner, demonstrating their versatility 19,20. A critical aspect of estrogen signaling is its reciprocal interaction with growth factor signaling. An *in vitro* study has shown that EGFR depletion can abolish the proliferative response and drug resistance of cancer cells to 17β-estradiol 21. In SNIP, previous research has identified ER positivity in 15% of cases 22. Additionally, dysregulated EGFR signaling, whether due to *EGFR* mutation 4,6,7 or the HPV-associated E5 oncoprotein 23, can play a central role in SNIP tumorigenesis. Based on our findings of increased expression of estrogen biosynthesis-related biomarkers and their positive correlation with EGFR expression, we propose that estrogen signaling may underlie the pathogenesis of SNIP.

Our analysis identified notable overexpression of three genes belonging to the Cytochrome P450 (CYP) superfamily in SNIP tissues, suggesting their involvement in key biological processes. The human genome encompasses 57 CYP genes across 18 families, primarily expressed in the liver 24,25. These enzymes are crucial for metabolizing exogenous substances such as drugs and xenobiotics, aiding in detoxification and preventing genomic damage 24,25. Specific CYPs convert all-trans-retinol into all-trans-retinoic acid, a vital component of vitamin A metabolism that affects the development and progression of inflammatory diseases and cancer 15,26. Additionally, CYPs play a significant role in the biosynthesis and oxidative metabolism of sex hormones 27. Research has shown that CYP polymorphisms are associated with altered risks for various diseases, including benign tumors 28. Although the specific mechanisms remain unclear, Bergheim *et al.* observed higher expression levels of certain CYPs in colon adenomas and disease-free controls compared to their adjacent normal tissues, suggesting their possible roles in tumorigenesis 29. Furthermore, Takeuchi *et al.* demonstrated that CYP2A5 can metabolically activate the carcinogen 4-(methylnitrosamino)-1-(3-pyridyl)-1-butanone (NNK), leading to adenoma formation in mouse lungs 30. These findings highlight the role of CYP enzymes in the development of benign tumors and support that CYPs might contribute to SNIP pathogenesis.

The Aldo-keto reductase (AKR) includes over 190 members across all phyla, with 15 AKR members identified in humans, predominantly from the AKR1 family 31. These enzymes are NADPH-dependent cytosolic proteins characterized by a unique (α/β)8-barrel motif that aids diverse biochemical functions, such as sex hormone biosynthesis and detoxification of aldehydes and ketones 31. Accumulating evidence has highlighted that certain AKRs modulate disease through both catalytic-dependent and -independent mechanisms 16. AKR1B10 typically shows downregulation in cancers and inflammatory conditions but plays an oncogenic role in tissues where it is not commonly expressed 32. Further research has associated AKR1B10 expression levels with acquired chemoresistance and survival outcomes in various cancers, uncovering the mechanisms involved 16,32. For instance, AKR1B10 may interact with glyceraldehyde-3-phosphate dehydrogenase to reduce autophagy by modifying its reductase activity in cancer cells 33. In benign neoplasms, AKR1B10 has been identified as a transcriptional target of p53 in colorectal adenomas, exhibiting decreased expression compared to normal controls 34. Our study demonstrated that AKR1B10 is upregulated in SNIP tissues, suggesting its potential role in the tumorigenesis of the sinonasal tract.

17β-Hydroxysteroid dehydrogenases (17β-HSDs) are vital oxidoreductases involved in the metabolism of estrogens and androgens. They catalyze NAD(P)H or NAD(P)+ dependent redox reactions at the 17β-position of the steroid, thus regulating steroid signaling 35,36. Among the fourteen identified mammal 17β-HSDs, specific subfamily members are implicated in various health conditions including breast and prostate cancer, and endometriosis 35,36. HSD17B13, a lipid droplet-associated enzyme 37, is upregulated in non-alcoholic fatty liver disease (NAFLD) 38. Genetic association studies have identified a protective HSD17B13 variant that confers decreased protein stability and reduced enzymatic activity 39, thereby mitigating progression from NAFLD to non-alcoholic steatohepatitis, liver fibrosis 17,39, and hepatocellular carcinoma 17. A recent structural analysis has elucidated how these protective variants disrupt the anchoring mechanisms of droplet-associated proteins to membranes, offering a therapeutic angle targeting HSD17B13 for these liver diseases 40. The identification of HSD17B13 as a dysregulated biomarker in SNIP highlights the broader significance of HSD17B13 in human health and diseases.

Therefore, targeting both estrogen signaling with selective estrogen receptor modulators (e.g., tamoxifen) 41 and estrogen biosynthesis with aromatase inhibitors (e.g., letrozole) 42 may offer a viable therapeutic strategy, given their established efficacy in estrogen-related neoplasms. Additionally, specific inhibitors targeting the overexpressed estrogen biosynthesis enzymes identified in SNIP, such as the five biomarkers from our study, may provide a more targeted approach by disrupting the altered downstream pathways contributing to SNIP development. Small-molecule inhibitors, including AKR1B10 inhibitors (e.g., statil, epalrestatl) 43 and CYP1B1 inhibitors (e.g., flavonoids, trans-stilbenes) 44, have shown preclinical efficacy in suppressing tumor growth. These findings suggest new avenues for therapeutic intervention in SNIP by targeting estrogen signaling.

### Study limitations

The primary limitation of our study is the small sample size used for the initial RNA-Seq analysis. This constraint was largely due to the availability of high-quality matched tissue samples and resource limitations. However, we addressed this limitation by comparing our RNA-Seq data with an independent gene expression dataset and subsequently validating our findings in a larger, independent cohort. Moreover, although these biomarkers are novel in the context of SNIP, their dysregulation have been reported in various neoplastic pathologies. Further enrichment and correlation analyses revealed their potential involvement in EGFR signaling, which is critical to SNIP pathogenesis, thereby supporting their significance. Nonetheless, future large-scale studies are warranted to further validate these biomarkers.

Another limitation is our limited understanding of the identified biomarkers' roles in the malignant transformation from SNIP to squamous cell carcinoma (IP-SCC). Although IP-SCC is relatively rare (5% to 10%), it has a poor prognosis, with five-year survival rates ranging from 39.6% to 65.7% 45. Due to this, significant efforts have been made to identify biomarkers that can predict malignant transformation. Long *et al.* identified *MSX2*, *PDCD4*, *KRAS* mutations, and *PTEN* as potential markers 45. In terms of EGFR signaling, *EGFR* mutations are frequently found in IP-SCC but are rare or absent 4-6 in *de novo* SCC, highlighting their distinct biological behaviors. EGFR plays a key oncogenic role in IP-SCC 4,5, as study has shown identical *EGFR* genotypes in SNIP and paired IP-SCC, with EGFR inhibitors proving effective in IP-SCC-derived cell lines 4. While our study identified distinct expression patterns of biomarkers related to the estrogen biosynthesis pathway between SNIP and SNSCC, further research is needed to clarify whether these markers contribute to IP-SCC.

## Conclusion

Taken together, our study identified biomarkers that were significantly differentially expressed in SNIP tissues compared with normal tissues across three independent cohorts. Their involvement in SNIP pathogenesis may be associated with the estrogen biosynthesis pathway. The AKR1B10, CYP2C19, and CYP3A5 protein expression levels were significantly different between patients with SNIP and SNSCC, whereas their expression levels in patients with SNSCC and controls were not significantly different. This specificity suggests that these three markers may be unique to SNIP rather than sinonasal malignancies. These findings highlight the diagnostic and therapeutic relevance of SNIP biomarkers and present them as potential targets for further diagnostic and therapeutic strategies specific to SNIP.

## Supplementary Material

Supplementary tables.

## Figures and Tables

**Table 1:** Demographic features and EGFR expression levels of 30 SNIP patients categorized by high and low biomarker expression subgroups

		AKR1B10			HSD17B3			CYP2C19			CYP1B1			CYP3A5		
		High (n = 18)	Low (n = 12)	p-value	High (n = 13)	Low (n = 17)	p-value	High (n = 19)	Low (n = 11)	p-value	High (n =16)	Low (n = 14)	p-value	High (n =15)	Low (n =15)	p-value
**Age (years)**				0.2098			1			0.104			1			1
	≥60	3 (16.7%)	5 (41.7%)		3 (23.1%)	5 (29.4%)		3 (15.8%)	5 (45.5%)		4 (25%)	4 (28.6%)		4 (26.7%)	5 (33.3%)	
	<60	15 (83.3%)	7 (58.3%)		10 (76.9%)	12 (70.6%)		16 (84.2%)	6 (54.5%)		12 (75%)	10 (71.4%)		11 (73.3%)	10 (66.7%)	
**Sex**				0.3575			0.0606			0.3717			0.3359			0.1686
	male	13 (72.2%)	11 (91.7%)		8 (61.5%)	16 (94.1%)		14 (73.7%)	10 (90.9%)		12 (75%)	13 (92.9%)		10 (66.7%)	14 (93.3%)	
	female	5 (27.8%)	1 (8.3%)		5 (38.5%)	1 (5.9%)		5 (26.3%)	1 (9.1%)		4 (25%)	1 (7.1%)		5 (33.3%)	1 (6.7%)	
**EGFR**		7189.04 ±1722.22	6018.22 ±2488.96	0.1556	7903.94 ±1265.29	5815.89 ±2195.26	0.0051*	7674.43 ±1506.90	5073.39 ±2010.74	0.0017*	7889.64 ±1349.30	5384.80 ±2043.37	0.0011*	8363.97 ±853.46	5077.45 ±1630.73	< 0.00001*

Abbreviations: EGFR = epidermal growth factor receptor; SNIP = sinonasal inverted papilloma. *p-values <0.05 are considered statistically significant.

## Abbreviations

SNIP: Sinonasal inverted papilloma
HPV: human papillomavirus
SCC: Squamous cell carcinoma
GLUT-1: glucose transporter 1
TERT: telomerase reverse transcriptase
GO: Gene Oncology
ICC: immunocytochemistry
AKR1B10: Aldo-Keto Reductase Family 1 Member B10
CYP1B1: Cytochrome P450 Family 1 Subfamily B Member 1
CYP2C19: Cytochrome P450 enzyme 2C19
CYP3A5: Cytochrome P450 Family 3 Subfamily A Member 5
HSD17B13: Hydroxysteroid 17-Beta Dehydrogenase 13
HSD17B2: Hydroxysteroid 17-Beta Dehydrogenase 2
HSD17B6: Hydroxysteroid 17-Beta Dehydrogenase 6
ERs: estrogen receptors
NNK: 4-(methylnitrosamino)-1-(3-pyridyl)-1-butanone
AKR: Aldo-keto reductase
17β-HSDs: 17β-Hydroxysteroid dehydrogenases
NAFLD: non-alcoholic fatty liver disease
IP-SCC: SNIP to squamous cell carcinoma
EGFR: Epidermal Growth Factor Receptor
MSX2: Msh Homeobox 2
PDCD4: Programmed Cell Death 4
KRAS: KRAS Proto-Oncogene, GTPase
PTEN: Phosphatase And Tensin Homolog
