# Supplementary material for: Identification and validation of Novel Estrogen Biosynthesis Biomarkers in Sinonasal Inverted Papilloma
Source: Int J Med Sci. 2025 Jan 1;22(1):158–69. doi: 10.7150/ijms.101753 (PMC11659834; doi:10.7150/ijms.101753)
Supplement: Supplementary file 1 — Supplementary tables. [file ijmsv22p0158s1.pdf]

Identification and validation of Novel Estrogen Biosynthesis Biomarkers in Sinonasal Inverted Papilloma

Type of article: Original Contribution

Author: Yi-Fang Yang, Sung-Huan Yu, Jia-Bin Liao, Yu-Hsuan Lin

Running title: Metabolic Biomarker for Inverted Papilloma

Numbers: Table-1; Figures-6; Supplementary Tables-4; Supplementary Material-3

Supplementary information

Supplementary Table 1	Related to Figure 2
Supplementary Table 2	Related to Figure 2
Supplementary Table 3	Related to Figure 2
Supplementary Table 4	Related to Figure 5 and Figure 6

Supplementary Material 1

Significantly altered genes with enzyme annotation in GSE270193

Supplementary Material 2

Significantly altered genes with enzyme annotation in GSE193016

Supplementary Material 3

The GeneOntology terms for biomarkers

Supplementary Table 1. Ingenuity Pathway Analysis (IPA) canonical pathways analysis of differentially expressed genes in SNIP tissues compared to normal tissues (GSE270193)

Ingenuity Canonical Pathways	p-value	Overlap	Molecules
Nicotine Degradation II	1.17×10^{-14}	17.9%	AOX1, CSGAL NACT1, CYP1B1, CYP2A6, CYP2C19, CYP2J2, CYP3A5, FMO1, FMO5, INMT, UGT1A9, UGT2A1
Nicotine Degradation III	3.65×10^{-12}	16.9%	AOX1, CSGAL NACT1, CYP1B1, CYP2A6, CYP2C19, CYP2J2, CYP3A5, FMO5, UGT1A9, UGT2A1
Estrogen Biosynthesis	1.15×10^{-11}	19.6%	AKR1B10, CYP1B1, CYP2A6, CYP2C19, CYP2J2, CYP3A5, HSD17B13, HSD17B2, HSD17B6
Relaxin Signaling	2.03×10^{-11}	8.4%	ADCY2, ADCY4, ADCY5, ADCY8, GNAZ, GUCY1A1, NOS3, NPR1, PDE11A, PDE2A, PDE3A, PDE5A, PDE7B
RAR Activation	3.34×10^{-11}	4.4%	ADCY2, ADCY4, ADCY5, ADCY8, ADH1B, ALDH1A2, GUCY1A1, HSD17B6, LRAT, PDE11A, PDE2A, PDE3A, PDE5A, PDE7B, RDH12, RHOJ, RHOV, SDR16C5, TGM2

Supplementary Table 2. IPA canonical pathways analysis of differentially expressed genes in SNIP tissues compared to normal tissues (GSE193016)

Ingenuity Canonical Pathways	p-value	Overlap	Molecules
LPS/IL-1 Mediated Inhibition of RXR Function	2.94×10^{-27}	14.8%	ACSBG1,ACSL6,ALDH1A2,ALDH1A3,ALDH1L2,ALDH3A1,ALDH3B1,CES2,CHST1,CHST2,CHST4,CYP2C18,CYP2C19,CYP2C9,CYP2S1,CYP2U1,CYP3A5,CYP3A7,FMO1,FMO3,FMO5,GSTM3,GSTM5,GSTO2,GSTT2/GSTT2B,HMGCS2,HS3ST1,HS3ST3A1,HS6ST2,HS6ST3,IL4I1,LIPC,MGST1,SOD3,SULT1C2,SULT1C4,SULT2B1,WSCD1
Xenobiotic Metabolism CAR Signaling Pathway	1.87×10^{-23}	16.0%	ALDH1A2,ALDH1A3,ALDH1L2,ALDH3A1,ALDH3B1,CHST1,CHST2,CHST4,CYP2C19,CYP2C9,CYP3A5,CYP3A7,FMO1,FMO3,FMO5,GSTM3,GSTM5,GSTO2,GSTT2/GSTT2B,HS3ST1,HS3ST3A1,HS6ST2,HS6ST3,MGST1,SOD3,SULT1C2,SULT1C4,SULT2B1,UGT1A9,UGT8,WSCD1
Xenobiotic Metabolism Signaling	5.51×10^{-23}	12.2%	ALDH1A2,ALDH1A3,ALDH1L2,ALDH3A1,ALDH3B1,CES2,CHST1,CHST2,CHST4,CYP1B1,CYP2C19,CYP2C9,CYP3A5,CYP3A7,FMO1,FMO3,FMO5,GSTM3,GSTM5,GSTO2,GSTT2/GSTT2B,HS3ST1,HS3ST3A1,HS6ST2,HS6ST3,IL4I1,MGST1,MRAS,RASD1,SOD3,SULT1C2,SULT1C4,SULT2B1,UGT1A9,UGT8,WSCD1
Xenobiotic Metabolism PXR Signaling Pathway	4.17×10^{-21}	14.9%	ALDH1A2,ALDH1A3,ALDH1L2,ALDH3A1,ALDH3B1,CES2,CHST1,CHST2,CHST4,CYP2C19,CYP2C9,CYP3A5,CYP3A7,GSTM3,GSTM5,GSTO2,GSTT2/GSTT2B,HS3ST1,HS3ST3A1,HS6ST2,HS6ST3,IL4I1,MGST1,SULT1C2,SULT1C4,SULT2B1,UGT1A9,UGT8,WSCD1
Estrogen Biosynthesis	3.77×10^{-20}	37.0%	AKR1B10,AKR1B15,AKR1C3,CYP1B1,CYP2C18,CYP2C19,CYP2C9,CYP2S1,CYP2U1,CYP3A5,CYP3A7,CYP4B1,CYP4F8,HSD17B1,HSD17B13,HSD17B2,HSD17B6

Supplementary Table 3. Overlapping differentially expressed genes between GSE270193 and GSE193016

Symbol	Gene Name
ACACB	acetyl-CoA carboxylase beta
ACSS3	acyl-CoA synthetase short chain family member 3
ADCY5	adenylate cyclase 5
ADCY8	adenylate cyclase 8
ADH1B	alcohol dehydrogenase 1B (class I), beta polypeptide
AKR1B10	aldo-keto reductase family 1 member B10
AKR1C1/AKR1C2	aldo-keto reductase family 1 member C2
ALDH1A2	aldehyde dehydrogenase 1 family member A2
ALOX15B	arachidonate 15-lipoxygenase type B
AOC3	amine oxidase copper containing 3
AOX1	aldehyde oxidase 1
APOBEC3A	apolipoprotein B mRNA editing enzyme catalytic subunit 3A
ASPA	aspartoacylase
B3GALT5	beta-1,3-galactosyltransferase 5
B3GNT8	UDP-GlcNAc:betaGal beta-1,3-N-acetylglucosaminyltransferase 8
CDO1	cysteine dioxygenase type 1
CEMIP	cell migration inducing hyaluronidase 1
CHI3L2	chitinase 3 like 2
COLGALT2	collagen beta(1-O)galactosyltransferase 2
CSGALNACT1	chondroitin sulfate N-acetylgalactosaminyltransferase 1
CYP1B1	cytochrome P450 family 1 subfamily B member 1
CYP26B1	cytochrome P450 family 26 subfamily B member 1
CYP27C1	cytochrome P450 family 27 subfamily C member 1
CYP2C19	cytochrome P450 family 2 subfamily C member 19
CYP3A5	cytochrome P450 family 3 subfamily A member 5
CYP4F3	cytochrome P450 family 4 subfamily F member 3
DIO2	iodothyronine deiodinase 2
DNASE1L3	deoxyribonuclease 1 like 3
DNM3	dynamin 3
DPYSL2	dihydropyrimidinase like 2
DPYSL3	dihydropyrimidinase like 3
DQX1	DEAQ-box RNA dependent ATPase 1
ESCO2	establishment of sister chromatid cohesion N-acetyltransferase 2
FADS3	fatty acid desaturase 3
FMO1	flavin containing dimethylaniline monoxygenase 1
FMO5	flavin containing dimethylaniline monoxygenase 5
GALNT13	polypeptide N-acetylgalactosaminyltransferase 13
GALNT15	polypeptide N-acetylgalactosaminyltransferase 15
GALNT16	polypeptide N-acetylgalactosaminyltransferase 16
GBP6	guanylate binding protein family member 6
GEM	GTP binding protein overexpressed in skeletal muscle
GIMAP7	GTPase, IMAP family member 7
GLT1D1	glycosyltransferase 1 domain containing 1
GLT8D2	glycosyltransferase 8 domain containing 2
GLYATL2	glycine-N-acyltransferase like 2
GNAZ	G protein subunit alpha z
GPAT2	glycerol-3-phosphate acyltransferase 2, mitochondrial
GPX2	glutathione peroxidase 2
GPX3	glutathione peroxidase 3
GSTM5	glutathione S-transferase mu 5
GUCY1A1	guanylate cyclase 1 soluble subunit alpha 1

HAO2	hydroxyacid oxidase 2
HMGCLL1	3-hydroxymethyl-3-methylglutaryl-CoA lyase L1
HPSE2	heparanase 2 (inactive)
HSD17B2	hydroxysteroid 17-beta dehydrogenase 2
HSD17B6	hydroxysteroid 17-beta dehydrogenase 6
HSD17B13	hydroxysteroid 17-beta dehydrogenase 13
HSD3B2	hydroxy-delta-5-steroid dehydrogenase, 3 beta- and steroid delta-isomerase 2
JPH2	junctophilin 2
LPO	lactoperoxidase
LYZ	lysozyme
MBOAT2	membrane bound O-acyltransferase domain containing 2
MOXD1	monooxygenase DBH like 1
MSRB3	methionine sulfoxide reductase B3
NEIL3	nei like DNA glycosylase 3
NLGN3	neuroligin 3
NPR1	natriuretic peptide receptor 1
PADI1	peptidyl arginine deiminase 1
PDCD1LG2	programmed cell death 1 ligand 2
PDE11A	phosphodiesterase 11A
PDE2A	phosphodiesterase 2A
PDE3A	phosphodiesterase 3A
PDE5A	phosphodiesterase 5A
PDZRN3	PDZ domain containing ring finger 3
PGM5	phosphoglucomutase 5
PLAAT1	phospholipase A and acyltransferase 1
PLCB4	phospholipase C beta 4
PLSCR4	phospholipid scramblase 4
PTGIS	prostaglandin I2 synthase
RAB38	RAB38, member RAS oncogene family
RAD51	RAD51 recombinase
RASD1	ras related dexamethasone induced 1
RASL12	RAS like family 12
RDH12	retinol dehydrogenase 12
RHOV	ras homolog family member V
RNASE1	ribonuclease A family member 1, pancreatic
SDC1	syndecan 1
SDR16C5	short chain dehydrogenase/reductase family 16C member 5
SOD3	superoxide dismutase 3
SPTSSB	serine palmitoyltransferase small subunit B
SRD5A1	steroid 5 alpha-reductase 1
SRD5A3	steroid 5 alpha-reductase 3
ST6GALNAC3	ST6 N-acetylgalactosaminide alpha-2,6-sialyltransferase 3
TNS2	tensin 2
TOP2A	DNA topoisomerase II alpha
UGT8	UDP glycosyltransferase 8
UGT1A9	UDP glucuronosyltransferase family 1 member A10
VNN1	vanin 1
VNN2	vanin 2

Supplementary Table 4. Demographic characteristics of control, SNIP, and SNCC specimens from a tissue microarray (NH1001a)

Group	Tissue ID.	Age	Sex	TNM	Stage
Control	Rns010047	46	M	-	-
Control	Rns010047	46	M	-	-
Control	Rns010074	14	M	-	-
Control	Rns010074	14	M	-	-
Control	Rns010112	17	M	-	-
Control	Rns010112	17	M	-	-
Control	Rns020713	69	M	-	-
Control	Rns020713	69	M	-	-
Control	Rns020883	74	M	-	-
Control	Rns020883	74	M	-	-
Control	Rns020886	40	M	-	-
Control	Rns020886	40	M	-	-
Control	Rns030211	42	M	-	-
Control	Rns030211	42	M	-	-
Control	Rns060310	25	F	-	-
Control	Rns060310	25	F	-	-
Control	Rns080080	62	M	-	-
Control	Rns080080	62	M	-	-
Control	Rns010110	66	M	-	-
Control	Rns010110	66	M	-	-
Control	Rns030767	40	M	-	-
Control	Rns030767	40	M	-	-
Control	Rns040229	65	M	-	-
Control	Rns040229	65	M	-	-
Control	Rns040832	51	F	-	-
Control	Rns040832	51	F	-	-
SNIP	Rns020263	43	M	-	-
SNIP	Rns020263	43	M	-	-
SNIP	Rns030061	30	M	-	-
SNIP	Rns030061	30	M	-	-
SNIP	Rns030248	42	M	-	-
SNIP	Rns030248	42	M	-	-
SNIP	Rns030251	52	M	-	-
SNIP	Rns030251	52	M	-	-
SNIP	Rns030711	73	M	-	-
SNIP	Rns030711	73	M	-	-
SNIP	Rns040029	68	F	-	-
SNIP	Rns040029	68	F	-	-
SNIP	Rns040119	50	M	-	-
SNIP	Rns040119	50	M	-	-
SNIP	Rns060009	55	M	-	-
SNIP	Rns060009	55	M	-	-
SNIP	Rns040393	43	M	-	-
SNIP	Rns040393	43	M	-	-
SNIP	Rns040408	62	M	-	-
SNIP	Rns040408	62	M	-	-
SNIP	Rns040886	56	M	-	-
SNIP	Rns040886	56	M	-	-
SNIP	Rns060037	37	F	-	-
SNIP	Rns060037	37	F	-	-
SNIP	Rns050425	71	F	-	-

SNIP	Rns050425	71	F	-	-
SNIP	Rns050440	54	M	-	-
SNIP	Rns050440	54	M	-	-
SNIP	Rns050441	34	M	-	-
SNIP	Rns050441	34	M	-	-
SNSCC	Rns020376	73	M	T1N0M0	I
SNSCC	Rns020376	73	M	T1N0M0	I
SNSCC	Rns020372	63	M	T1N0M0	I
SNSCC	Rns020372	63	M	T1N0M0	I
SNSCC	Rns070100	44	M	T2N1M0	II
SNSCC	Rns070100	44	M	T2N1M0	II
SNSCC	Rns050351	74	M	T2N0M0	II
SNSCC	Rns050351	74	M	T2N0M0	II
SNSCC	Rns050352	58	M	T1N0M0	I
SNSCC	Rns050352	58	M	T1N0M0	I
SNSCC	Rns110055	58	M	T2N0M0	II
SNSCC	Rns110055	58	M	T2N0M0	II
SNSCC	Rns070144	50	M	T2N0M0	II
SNSCC	Rns070144	50	M	T2N0M0	II
SNSCC	Rns040787	56	M	T1N0M0	I
SNSCC	Rns040787	56	M	T1N0M0	I
SNSCC	Rns050268	45	F	T1N0M0	I
SNSCC	Rns050268	45	F	T1N0M0	I
SNSCC	Rns080044	58	M	T1N0M0	I
SNSCC	Rns080044	58	M	T1N0M0	I
SNSCC	Rns020666	43	M	T3N0M0	III
SNSCC	Rns020666	43	M	T3N0M0	III
SNSCC	Rns020614	47	M	T2N0M0	II
SNSCC	Rns020614	47	M	T2N0M0	II
SNSCC	Rns040303	41	M	T2N0M0	II
SNSCC	Rns040303	41	M	T2N0M0	II
SNSCC	Rns050047	51	F	T2N0M0	II
SNSCC	Rns050047	51	F	T2N0M0	II
SNSCC	Rns070030	28	M	T2N0M0	II
SNSCC	Rns070030	28	M	T2N0M0	II
SNSCC	Rns010007	73	M	T4AN0M0	IV
SNSCC	Rns030046	46	M	T1N0M0	I
SNSCC	Rns030046	46	M	T1N0M0	I

Supplementary Material 1. Significantly altered genes with enzyme annotation in GSE270193

Symbol	Expr Log Ratio	Type(s)
ACACB	2.186	enzyme
ACSS3	2.097	enzyme
ADCY2	3.043	enzyme
ADCY4	2.739	enzyme
ADCY5	2.598	enzyme
ADCY8	4.647	enzyme
ADH1B	2.011	enzyme
AKR1B10	-4.821	enzyme
AKR1C1/AKR1C2	-2.398	enzyme
ALDH1A2	4.448	enzyme
ALDH3B2	-2.648	enzyme
ALOX15B	-3.641	enzyme
AMY1C (includes others)	3.875	enzyme
AOC3	4.616	enzyme
AOX1	4.295	enzyme
APOBEC3A	-4.274	enzyme
ARSL	2.831	enzyme
ASPA	2.866	enzyme
B3GALT5	-3.512	enzyme
B3GNT8	-4.517	enzyme
B4GALNT4	2.468	enzyme
CDO1	3.719	enzyme
CEMIP	-2.018	enzyme
CES1	2.698	enzyme
CH25H	2.864	enzyme
CHI3L2	3.582	enzyme
CMBL	2.203	enzyme
COLGALT2	2.282	enzyme
CSGALNACT1	2.329	enzyme
CYP11A1	6.941	enzyme
CYP1B1	3.485	enzyme
CYP26B1	3.8	enzyme
CYP27C1	-4.395	enzyme
CYP2A6	2.981	enzyme
CYP2C19	-4.067	enzyme
CYP2J2	2.985	enzyme
CYP3A5	-4.434	enzyme
CYP4F3	-2.437	enzyme
DCT	3.998	enzyme
DIO2	2.158	enzyme
DIRAS2	4.703	enzyme
DNASE1L3	3.601	enzyme
DNM3	3.1	enzyme
DPYS	10.147	enzyme
DPYSL2	2.197	enzyme
DPYSL3	3.442	enzyme
DQX1	-4.086	enzyme
DSEL	2.36	enzyme
DUOX2	-2.547	enzyme
ECI2	3.074	enzyme
ENPP5	2.449	enzyme

ESCO2	-2.112	enzyme
FA2H	-3.332	enzyme
FADS2	2.011	enzyme
FADS3	2.43	enzyme
FKBP10	2.316	enzyme
FMO1	2.677	enzyme
FMO5	2.107	enzyme
GALNT13	2.704	enzyme
GALNT15	3.617	enzyme
GALNT16	2.594	enzyme
GBP6	-2.125	enzyme
GCNT3	-3.049	enzyme
GEM	2.443	enzyme
GGT7	2.017	enzyme
GIMAP7	2.669	enzyme
GLT1D1	-3.525	enzyme
GLT8D2	2.661	enzyme
GLYATL2	7.01	enzyme
GNAZ	3.622	enzyme
GPAT2	6.263	enzyme
GPX2	-2.058	enzyme
GPX3	2.435	enzyme
GPX6	6.525	enzyme
GSTA2	5.143	enzyme
GSTM5	5.297	enzyme
GUCY1A1	2.58	enzyme
GXYLT2	2.043	enzyme
HAL	-3.287	enzyme
HAO2	4.295	enzyme
HMGA2	-2.641	enzyme
HMGCLL1	4.846	enzyme
HPSE2	3.794	enzyme
HSD17B2	2.045	enzyme
HSD17B6	7.006	enzyme
HSD17B13	4.491	enzyme
HSD3B2	7.689	enzyme
HSPG2	2.027	enzyme
INMT	3.358	enzyme
JPH2	3.432	enzyme
KYNU	-2.887	enzyme
LAMA4	2.253	enzyme
LIPK	-6.219	enzyme
LPO	7.091	enzyme
LRAT	-4.193	enzyme
LYZ	7.333	enzyme
MAGI2	2.022	enzyme
MBOAT2	-2.691	enzyme
MOXD1	4.24	enzyme
MSRB3	3.567	enzyme
NEIL3	-2.856	enzyme
NLGN3	3.124	enzyme
NOS3	2.637	enzyme
NPR1	3.327	enzyme
PADI1	-2.721	enzyme

PDCD1LG2	2.725	enzyme
PDE11A	4.651	enzyme
PDE2A	3.689	enzyme
PDE3A	3.63	enzyme
PDE5A	2.424	enzyme
PDE7B	2.463	enzyme
PDZRN3	3.684	enzyme
PGM5	6.869	enzyme
PLA1A	3.659	enzyme
PLAAT1	-4.675	enzyme
PLCB4	2.093	enzyme
PLSCR4	2.284	enzyme
PTGIS	4.767	enzyme
PYGM	3.069	enzyme
RAB38	-2.086	enzyme
RAB6B	3.703	enzyme
RAD51	-3.739	enzyme
RASD1	2.506	enzyme
RASL12	4.488	enzyme
RDH12	-5.694	enzyme
RHOJ	2.63	enzyme
RHOV	-2.174	enzyme
RNASE1	2.927	enzyme
RNF122	2.064	enzyme
SDC1	-2.166	enzyme
SDR16C5	-2.394	enzyme
SEPTIN4	2.633	enzyme
SHOC1	-3.125	enzyme
SOD3	4.591	enzyme
SPTSSB	-3.598	enzyme
SRD5A1	-2.138	enzyme
SRD5A3	-2.072	enzyme
ST6GAL2	2.569	enzyme
ST6GALNAC3	2.167	enzyme
SULF1	2.191	enzyme
TGM2	2.246	enzyme
TGM5	-2.772	enzyme
TMOD1	3.949	enzyme
TNS2	2.751	enzyme
TOP2A	-2.076	enzyme
TRIM58	3.201	enzyme
UGT8	2.212	enzyme
UGT1A9	-4.068	enzyme
UGT2A1	2.652	enzyme
UGT3A2	4.52	enzyme
VNN1	-2.105	enzyme
VNN2	-2.541	enzyme

Supplementary Material 2. Significantly altered genes with enzyme annotation in GSE193016

Symbol	Expr Log Ratio	Type(s)
AADAC	3.13	enzyme
ABHD17C	2.04	enzyme
ACACB	-2.5	enzyme
ACADL	-3.82	enzyme
ACAT1	-2.54	enzyme
ACOT11	2.65	enzyme
ACSBG1	3.31	enzyme
ACSL6	-3.09	enzyme
ACSS3	-3.81	enzyme
ACTC1	-2.41	enzyme
ADCY1	-3.65	enzyme
ADCY5	-6.88	enzyme
ADCY8	-3	enzyme
ADH7	5.39	enzyme
ADH1B	-3.9	enzyme
AGBL2	2.51	enzyme
AKR1B10	6.91	enzyme
AKR1B15	5.88	enzyme
AKR1C3	2.32	enzyme
AKR1C1/AKR1C2	3.63	enzyme
AKR1E2	-2.07	enzyme
ALDH1A2	-4.44	enzyme
ALDH1A3	-2.15	enzyme
ALDH1L2	-3.53	enzyme
ALDH3A1	3.69	enzyme
ALDH3B1	2.1	enzyme
ALOX15	4.26	enzyme
ALOX15B	4.27	enzyme
AMDHD1	-2.22	enzyme
ANXA1	2.49	enzyme
AOC3	-4.88	enzyme
AOX1	-4.35	enzyme
APOBEC3A	3.82	enzyme
ARL4D	2.27	enzyme
ART3	-7.88	enzyme
ART5	-3.9	enzyme
ASPA	-3.76	enzyme
ASPG	4.73	enzyme
ASRGL1	-2.25	enzyme
ATP6V0E2	-2.57	enzyme
B3GALNT1	-2.33	enzyme
B3GALT5	2.75	enzyme
B3GAT1	-3.44	enzyme
B3GNT8	2.25	enzyme
B4GALT5	2.28	enzyme
BBOX1	2.35	enzyme
BCHE	-2.36	enzyme
BHMT2	-3.91	enzyme
C10orf90	-2.61	enzyme
CA2	-5.67	enzyme
CA3	-2.75	enzyme

CA12	2.14	enzyme
CBLC	3.38	enzyme
CBS/LOC102724560	-5.32	enzyme
CDO1	-5.05	enzyme
CEMIP	3.48	enzyme
CERS1	-2.57	enzyme
CES2	2.18	enzyme
CHD5	-2.99	enzyme
CHI3L1	2.04	enzyme
CHI3L2	-5.88	enzyme
CHST1	-2.34	enzyme
CHST2	-2.84	enzyme
CHST4	2.75	enzyme
CHST6	2.71	enzyme
CHST8	-3.97	enzyme
CLC	2.08	enzyme
CLIC2	-2.12	enzyme
COLGALT2	-5.57	enzyme
COX6B2	3.05	enzyme
COX7A1	-3.14	enzyme
CP	3.08	enzyme
CRACR2A	-2.29	enzyme
CSGALNACT1	-3.56	enzyme
CSMD3	2.08	enzyme
CTH	-2.93	enzyme
CYP1B1	-5.02	enzyme
CYP24A1	2.76	enzyme
CYP26A1	3.36	enzyme
CYP26B1	-2.02	enzyme
CYP27A1	-2.79	enzyme
CYP27C1	2.84	enzyme
CYP2C9	5.93	enzyme
CYP2C18	4.34	enzyme
CYP2C19	4.09	enzyme
CYP2S1	3.55	enzyme
CYP2U1	-2.11	enzyme
CYP3A5	4.18	enzyme
CYP3A7	2.13	enzyme
CYP3A43	4.03	enzyme
CYP4B1	4.94	enzyme
CYP4F3	2.26	enzyme
CYP4F8	3.11	enzyme
CYP4F11	2.52	enzyme
CYP4F12	2.67	enzyme
CYP4F22	3.47	enzyme
DDX25	-2.07	enzyme
DEGS2	2.37	enzyme
DHRS9	4.27	enzyme
DIAPH3	2.87	enzyme
DIO1	3.17	enzyme
DIO2	-3.36	enzyme
DIO3	-3.49	enzyme
DIRAS1	-4.13	enzyme
DIRAS3	-4.44	enzyme

DNAH3	3.27	enzyme
DNASE1L3	-2.65	enzyme
DNM3	-2.25	enzyme
DPYSL2	-2.63	enzyme
DPYSL3	-4.08	enzyme
DPYSL4	-2.7	enzyme
DQX1	4.55	enzyme
DUOX1	2.69	enzyme
DUSP26	-3.08	enzyme
ENPP1	-3.57	enzyme
ENPP2	-2.09	enzyme
ENPP3	-4.57	enzyme
ENPP6	-2.4	enzyme
EPHX3	4.85	enzyme
ERCC6L	3.49	enzyme
ESCO2	2.45	enzyme
ETHE1	2.82	enzyme
EXO1	2.61	enzyme
FADS3	-2.93	enzyme
FAR2	-2.23	enzyme
FBXL13	-2.03	enzyme
FBXO27	2.1	enzyme
FDXR	2.57	enzyme
FKBP7	-2.42	enzyme
FKBP11	-2.06	enzyme
FMO1	-4.3	enzyme
FMO3	-3.73	enzyme
FMO5	-3.18	enzyme
FTCD	-2.54	enzyme
GAD1	-2.31	enzyme
GALNT6	-4.61	enzyme
GALNT13	-5.57	enzyme
GALNT14	3.78	enzyme
GALNT15	-6.33	enzyme
GALNT16	-3.5	enzyme
GBP6	4.62	enzyme
GCLM	2.33	enzyme
GDPD2	2.73	enzyme
GDPD5	-3.01	enzyme
GEM	-3.02	enzyme
GFPT2	-2.36	enzyme
GGT5	-3.27	enzyme
GIMAP7	-2.56	enzyme
GLT1D1	3.06	enzyme
GLT8D2	-2.01	enzyme
GLYATL2	-7.66	enzyme
GM2A	2.26	enzyme
GMPR	-2.5	enzyme
GNA15	3.8	enzyme
GNAO1	-2.74	enzyme
GNAZ	-4.11	enzyme
GNMT	-2.86	enzyme
GPAT2	-4.98	enzyme
GPD1	-2.76	enzyme

GPX2	4.78	enzyme
GPX3	-2.49	enzyme
GSTM3	-3.29	enzyme
GSTM5	-3.54	enzyme
GSTO2	-4.78	enzyme
GSTT2/GSTT2B	-2.74	enzyme
GUCY1A1	-2.57	enzyme
GYG2	-2.38	enzyme
HAO2	-4.81	enzyme
HAS2	2.45	enzyme
HAS3	3.94	enzyme
HMGCLL1	-4.43	enzyme
HMGCS2	-4.78	enzyme
HPSE2	-5.46	enzyme
HS3ST1	2.46	enzyme
HS3ST3A1	-2.58	enzyme
HS6ST2	6.29	enzyme
HS6ST3	-6.04	enzyme
HSD11B1	-3.61	enzyme
HSD17B1	2.1	enzyme
HSD17B2	-2.43	enzyme
HSD17B6	-5.31	enzyme
HSD17B13	-2.56	enzyme
HSD3B2	-2.12	enzyme
IL4I1	2.03	enzyme
IYD	2.2	enzyme
JPH2	-5.1	enzyme
KIF14	3.12	enzyme
KIF18A	2.73	enzyme
KIF5C	-2.49	enzyme
KIFC1	2.33	enzyme
LIPC	-2.32	enzyme
LMO7	2	enzyme
LPL	-2.14	enzyme
LPO	-13	enzyme
LYZ	-6.52	enzyme
MAGEL2	-3.99	enzyme
MBOAT2	2.5	enzyme
MCM4	2.33	enzyme
MGAM	2.1	enzyme
MGAT5B	-2.43	enzyme
MGST1	3.14	enzyme
MOGAT1	-4.93	enzyme
MOXD1	-3.28	enzyme
MPO	-2.41	enzyme
MRAS	-3.05	enzyme
MSRB3	-3.75	enzyme
MYH14	2.52	enzyme
NAT2	-2.48	enzyme
NEIL3	3.73	enzyme
NEU4	4.62	enzyme
NEURL3	-2.23	enzyme
NLGN3	-3.46	enzyme
NOS1	-2.31	enzyme

NOX1	2.13	enzyme
NPR1	-3.09	enzyme
NUGGC	-2.44	enzyme
OLAH	-4.66	enzyme
OTUB2	2.11	enzyme
PADI1	8.24	enzyme
PADI2	-3.47	enzyme
PADI3	2.01	enzyme
PDCD1LG2	-2.21	enzyme
PDE11A	-7.12	enzyme
PDE1A	-3.56	enzyme
PDE1B	-2.44	enzyme
PDE2A	-3.73	enzyme
PDE3A	-4.26	enzyme
PDE3B	-2.84	enzyme
PDE4B	-2.32	enzyme
PDE4C	2.97	enzyme
PDE5A	-3.84	enzyme
PDE8B	-2.75	enzyme
PDZRN3	-3.72	enzyme
PGM2	2.27	enzyme
PGM5	-7.32	enzyme
PHGDH	-2.26	enzyme
PITPNM3	2.46	enzyme
PIWIL1	2.11	enzyme
PLA2G5	-5.59	enzyme
PLA2G7	2.99	enzyme
PLA2G2A	-6.99	enzyme
PLA2G4E	2.81	enzyme
PLA2G4F	3.14	enzyme
PLAAT1	2.01	enzyme
PLBD1	2.06	enzyme
PLCB1	-3.9	enzyme
PLCB4	-3.16	enzyme
PLCH2	2.17	enzyme
PLCL2	-2.07	enzyme
PLSCR4	-2.44	enzyme
PNLDC1	2.28	enzyme
PNPLA1	2.62	enzyme
POLQ	2.87	enzyme
PPIL6	2.34	enzyme
PRODH	3.78	enzyme
PTGDS	-2.59	enzyme
PTGIS	-5.63	enzyme
PYCR1	-2.14	enzyme
QPRT	-2.22	enzyme
RAB25	2.53	enzyme
RAB30	-2.12	enzyme
RAB38	3.89	enzyme
RAB3C	-3.26	enzyme
RAB9B	-3.2	enzyme
RAD51	2.91	enzyme
RAD51B	-3.47	enzyme
RAD54L	2.68	enzyme

RASD1	-3.09	enzyme
RASL12	-4.05	enzyme
RASL10B	-2.61	enzyme
RASL11B	-3.07	enzyme
RDH10	3.17	enzyme
RDH12	4.52	enzyme
RECQL4	2.16	enzyme
RENBP	-2.22	enzyme
RERG	2.09	enzyme
RGN	-2.98	enzyme
RGS4	-2.71	enzyme
RGS5	-2.37	enzyme
RGS12	2.02	enzyme
RGS20	4.53	enzyme
RHOBTB3	-2.13	enzyme
RHOV	4.16	enzyme
RNASE1	-2.72	enzyme
RNASE4	-2.08	enzyme
RNASE8	-4.01	enzyme
RNF148	-2.19	enzyme
RNF180	-3.8	enzyme
RNF182	-3.61	enzyme
RNF183	2.75	enzyme
RRM2	4.01	enzyme
SAT1	2.1	enzyme
SDC1	2.1	enzyme
SDR16C5	3.58	enzyme
SDS	2.94	enzyme
SETD9	2.22	enzyme
SH3GL2	-2.54	enzyme
SH3RF2	3.14	enzyme
SOD3	-5.01	enzyme
SPTSSB	2.44	enzyme
SRD5A1	3.33	enzyme
SRD5A2	3.87	enzyme
SRD5A3	2.18	enzyme
ST3GAL5	-2.88	enzyme
ST3GAL6	-2.54	enzyme
ST6GALNAC1	3.24	enzyme
ST6GALNAC3	-3.9	enzyme
SULT1C2	-3.36	enzyme
SULT1C4	-3.38	enzyme
SULT2B1	3.13	enzyme
TDRD9	-2.74	enzyme
TENT5A	-2.27	enzyme
TENT5C	-3.18	enzyme
TGM1	3.66	enzyme
TH	-2.27	enzyme
TKT	2.38	enzyme
TKTL1	-4.83	enzyme
TNNI2	2.68	enzyme
TNS2	-2.48	enzyme
TOP2A	3.83	enzyme
TP53I3	2	enzyme

TRIM9	-3.56	enzyme
TRIM31	4.21	enzyme
TRIM36	-2.3	enzyme
TRIM59	2	enzyme
TRIM63	-3.64	enzyme
TRMT9B	-2.16	enzyme
TTLL6	2.67	enzyme
TTLL7	-3.59	enzyme
TULP2	-2.12	enzyme
TXN	2.23	enzyme
TXNDC17	2.33	enzyme
TYMS	3.01	enzyme
TYMSOS	2.58	enzyme
UBASH3B	2.84	enzyme
UBE2C	3.61	enzyme
UBE2T	2.78	enzyme
UGT8	-2.15	enzyme
UGT1A9 (includes others)	4.95	enzyme
UNC80	-4.95	enzyme
VAT1L	-5.85	enzyme
VNN1	2.54	enzyme
VNN2	2.61	enzyme
VNN3P	3.14	enzyme
WSCD1	-3.22	enzyme
ZDHHC15	-2.98	enzyme

Supplementary Material 3. The GeneOntology terms for biomarkers

**biological process**		
CYP1B1	GO:0000038	very long-chain fatty acid metabolic process
CYP2C19	GO:0000038	very long-chain fatty acid metabolic process
CYP1B1	GO:0000302	response to reactive oxygen species
AKR1B10	GO:0001523	retinoid metabolic process
CYP1B1	GO:0001523	retinoid metabolic process
CYP1B1	GO:0001525	angiogenesis
CYP1B1	GO:0001817	regulation of cytokine production
CYP1B1	GO:0001819	positive regulation of cytokine production
CYP1B1	GO:0003008	system process
AKR1B10	GO:0006066	alcohol metabolic process
CYP1B1	GO:0006066	alcohol metabolic process
AKR1B10	GO:0006081	cellular aldehyde metabolic process
CYP1B1	GO:0006081	cellular aldehyde metabolic process
CYP1B1	GO:0006082	organic acid metabolic process
CYP2C19	GO:0006082	organic acid metabolic process
CYP1B1	GO:0006355	regulation of transcription, DNA-dependent
AKR1B10	GO:0006629	lipid metabolic process
CYP1B1	GO:0006629	lipid metabolic process
CYP2C19	GO:0006629	lipid metabolic process
CYP3A5	GO:0006629	lipid metabolic process
CYP1B1	GO:0006631	fatty acid metabolic process
CYP2C19	GO:0006631	fatty acid metabolic process
CYP1B1	GO:0006643	membrane lipid metabolic process
CYP1B1	GO:0006690	icosanoid metabolic process
CYP2C19	GO:0006690	icosanoid metabolic process
AKR1B10	GO:0006714	sesquiterpenoid metabolic process
AKR1B10	GO:0006720	isoprenoid metabolic process
CYP1B1	GO:0006720	isoprenoid metabolic process
CYP2C19	GO:0006720	isoprenoid metabolic process
AKR1B10	GO:0006721	terpenoid metabolic process
CYP1B1	GO:0006721	terpenoid metabolic process
CYP2C19	GO:0006721	terpenoid metabolic process
CYP1B1	GO:0006725	cellular aromatic compound metabolic process
CYP3A5	GO:0006730	one-carbon metabolic process
CYP1B1	GO:0006766	vitamin metabolic process
CYP1B1	GO:0006775	fat-soluble vitamin metabolic process
CYP1B1	GO:0006776	vitamin A metabolic process
CYP1B1	GO:0006805	xenobiotic metabolic process
CYP2C19	GO:0006805	xenobiotic metabolic process
CYP3A5	GO:0006805	xenobiotic metabolic process

CYP1B1	GO:0006807	nitrogen compound metabolic process
CYP3A5	GO:0006807	nitrogen compound metabolic process
CYP1B1	GO:0006809	nitric oxide biosynthetic process
CYP1B1	GO:0006917	induction of apoptosis
CYP1B1	GO:0006928	cellular component movement
CYP1B1	GO:0006950	response to stress
CYP1B1	GO:0006979	response to oxidative stress
CYP1B1	GO:0007155	cell adhesion
CYP1B1	GO:0007162	negative regulation of cell adhesion
AKR1B10	GO:0007165	signal transduction
AKR1B10	GO:0007586	digestion
CYP1B1	GO:0007600	sensory perception
CYP1B1	GO:0007601	visual perception
AKR1B10	GO:0007602	phototransduction
AKR1B10	GO:0007603	phototransduction, visible light
AKR1B10	GO:0008152	metabolic process
CYP1B1	GO:0008152	metabolic process
CYP2C19	GO:0008152	metabolic process
CYP3A5	GO:0008152	metabolic process
AKR1B10	GO:0008202	steroid metabolic process
CYP1B1	GO:0008202	steroid metabolic process
CYP2C19	GO:0008202	steroid metabolic process
CYP3A5	GO:0008202	steroid metabolic process
CYP1B1	GO:0008210	estrogen metabolic process
CYP1B1	GO:0008285	negative regulation of cell proliferation
AKR1B10	GO:0008300	isoprenoid catabolic process
CYP1B1	GO:0008629	induction of apoptosis by intracellular signals
CYP1B1	GO:0008631	induction of apoptosis by oxidative stress
AKR1B10	GO:0009056	catabolic process
CYP1B1	GO:0009056	catabolic process
CYP2C19	GO:0009056	catabolic process
CYP3A5	GO:0009056	catabolic process
CYP1B1	GO:0009058	biosynthetic process
AKR1B10	GO:0009314	response to radiation
CYP1B1	GO:0009404	toxin metabolic process
AKR1B10	GO:0009416	response to light stimulus
AKR1B10	GO:0009581	detection of external stimulus
AKR1B10	GO:0009582	detection of abiotic stimulus
AKR1B10	GO:0009583	detection of light stimulus
AKR1B10	GO:0009584	detection of visible light
AKR1B10	GO:0009605	response to external stimulus
AKR1B10	GO:0009628	response to abiotic stimulus

CYP1B1	GO:0009636	response to toxin
CYP1B1	GO:0009653	anatomical structure morphogenesis
CYP3A5	GO:0009820	alkaloid metabolic process
CYP3A5	GO:0009822	alkaloid catabolic process
CYP1B1	GO:0009889	regulation of biosynthetic process
HSD17B13	GO:0009889	regulation of biosynthetic process
HSD17B13	GO:0009891	positive regulation of biosynthetic process
HSD17B13	GO:0009893	positive regulation of metabolic process
CYP1B1	GO:0009966	regulation of signal transduction
CYP1B1	GO:0009967	positive regulation of signal transduction
AKR1B10	GO:0009987	cellular process
CYP1B1	GO:0009987	cellular process
CYP2C19	GO:0009987	cellular process
CYP3A5	GO:0009987	cellular process
CYP1B1	GO:0010033	response to organic substance
CYP1B1	GO:0010035	response to inorganic substance
CYP1B1	GO:0010468	regulation of gene expression
CYP1B1	GO:0010556	regulation of macromolecule biosynthetic process
CYP1B1	GO:0010574	regulation of vascular endothelial growth factor production
CYP1B1	GO:0010575	positive regulation vascular endothelial growth factor production
CYP1B1	GO:0010627	regulation of intracellular protein kinase cascade
CYP1B1	GO:0010646	regulation of cell communication
CYP1B1	GO:0010647	positive regulation of cell communication
CYP1B1	GO:0010740	positive regulation of intracellular protein kinase cascade
CYP1B1	GO:0010817	regulation of hormone levels
CYP1B1	GO:0010941	regulation of cell death
CYP1B1	GO:0010942	positive regulation of cell death
CYP1B1	GO:0012502	induction of programmed cell death
CYP1B1	GO:0014070	response to organic cyclic compound
AKR1B10	GO:0016042	lipid catabolic process
CYP1B1	GO:0016042	lipid catabolic process
CYP1B1	GO:0016043	cellular component organization
CYP2C19	GO:0016098	monoterpenoid metabolic process
AKR1B10	GO:0016101	diterpenoid metabolic process
CYP1B1	GO:0016101	diterpenoid metabolic process
AKR1B10	GO:0016107	sesquiterpenoid catabolic process
AKR1B10	GO:0016115	terpenoid catabolic process
CYP1B1	GO:0016125	sterol metabolic process
CYP1B1	GO:0016337	cell-cell adhesion
CYP1B1	GO:0016477	cell migration
AKR1B10	GO:0016487	farnesol metabolic process
AKR1B10	GO:0016488	farnesol catabolic process

CYP2C19	GO:0017144	drug metabolic process
CYP3A5	GO:0017144	drug metabolic process
HSD17B13	GO:0019216	regulation of lipid metabolic process
CYP1B1	GO:0019219	regulation of nucleobase-containing compound metabolic process
CYP1B1	GO:0019222	regulation of metabolic process
HSD17B13	GO:0019222	regulation of metabolic process
CYP1B1	GO:0019369	arachidonic acid metabolic process
CYP2C19	GO:0019369	arachidonic acid metabolic process
CYP1B1	GO:0019373	epoxygenase P450 pathway
CYP2C19	GO:0019373	epoxygenase P450 pathway
CYP1B1	GO:0019748	secondary metabolic process
CYP1B1	GO:0019752	carboxylic acid metabolic process
CYP2C19	GO:0019752	carboxylic acid metabolic process
CYP1B1	GO:0022603	regulation of anatomical structure morphogenesis
CYP1B1	GO:0022610	biological adhesion
CYP1B1	GO:0023051	regulation of signaling
CYP1B1	GO:0023056	positive regulation of signaling
CYP1B1	GO:0030155	regulation of cell adhesion
CYP1B1	GO:0030198	extracellular matrix organization
CYP1B1	GO:0030199	collagen fibril organization
CYP1B1	GO:0030334	regulation of cell migration
CYP1B1	GO:0030336	negative regulation of cell migration
CYP1B1	GO:0031323	regulation of cellular metabolic process
CYP1B1	GO:0031326	regulation of cellular biosynthetic process
CYP1B1	GO:0032088	negative regulation of NF-kappaB transcription factor activity
AKR1B10	GO:0032501	multicellular organismal process
CYP1B1	GO:0032501	multicellular organismal process
CYP1B1	GO:0032502	developmental process
CYP1B1	GO:0032787	monocarboxylic acid metabolic process
CYP2C19	GO:0032787	monocarboxylic acid metabolic process
CYP1B1	GO:0032879	regulation of localization
CYP1B1	GO:0033554	cellular response to stress
CYP1B1	GO:0033559	unsaturated fatty acid metabolic process
CYP2C19	GO:0033559	unsaturated fatty acid metabolic process
CYP1B1	GO:0033628	regulation of cell adhesion mediated by integrin
CYP1B1	GO:0033629	negative regulation of cell adhesion mediated by integrin
AKR1B10	GO:0034308	primary alcohol metabolic process
AKR1B10	GO:0034310	primary alcohol catabolic process
CYP1B1	GO:0034599	cellular response to oxidative stress
CYP1B1	GO:0034614	cellular response to reactive oxygen species
CYP1B1	GO:0034641	cellular nitrogen compound metabolic process
CYP3A5	GO:0034641	cellular nitrogen compound metabolic process

CYP1B1	GO:0034754	cellular hormone metabolic process
CYP1B1	GO:0040011	locomotion
CYP1B1	GO:0040012	regulation of locomotion
CYP1B1	GO:0040013	negative regulation of locomotion
CYP1B1	GO:0042127	regulation of cell proliferation
CYP1B1	GO:0042180	cellular ketone metabolic process
CYP2C19	GO:0042180	cellular ketone metabolic process
AKR1B10	GO:0042214	terpene metabolic process
CYP1B1	GO:0042221	response to chemical stimulus
CYP1B1	GO:0042445	hormone metabolic process
CYP1B1	GO:0042542	response to hydrogen peroxide
CYP1B1	GO:0042572	retinol metabolic process
CYP1B1	GO:0042574	retinal metabolic process
CYP2C19	GO:0042737	drug catabolic process
CYP3A5	GO:0042737	drug catabolic process
CYP2C19	GO:0042738	exogenous drug catabolic process
CYP1B1	GO:0042981	regulation of apoptosis
CYP1B1	GO:0043062	extracellular structure organization
CYP1B1	GO:0043065	positive regulation of apoptosis
CYP1B1	GO:0043067	regulation of programmed cell death
CYP1B1	GO:0043068	positive regulation of programmed cell death
CYP1B1	GO:0043288	apocarotenoid metabolic process
CYP1B1	GO:0043433	negative regulation of sequence-specific DNA binding transcription factor activity
CYP1B1	GO:0043436	oxoacid metabolic process
CYP2C19	GO:0043436	oxoacid metabolic process
CYP1B1	GO:0043542	endothelial cell migration
CYP1B1	GO:0044092	negative regulation of molecular function
AKR1B10	GO:0044237	cellular metabolic process
CYP1B1	GO:0044237	cellular metabolic process
CYP2C19	GO:0044237	cellular metabolic process
CYP3A5	GO:0044237	cellular metabolic process
AKR1B10	GO:0044238	primary metabolic process
CYP1B1	GO:0044238	primary metabolic process
CYP2C19	GO:0044238	primary metabolic process
CYP3A5	GO:0044238	primary metabolic process
AKR1B10	GO:0044242	cellular lipid catabolic process
CYP1B1	GO:0044242	cellular lipid catabolic process
AKR1B10	GO:0044248	cellular catabolic process
CYP1B1	GO:0044248	cellular catabolic process
CYP2C19	GO:0044248	cellular catabolic process
CYP3A5	GO:0044248	cellular catabolic process
CYP1B1	GO:0044249	cellular biosynthetic process

AKR1B10	GO:0044255	cellular lipid metabolic process
CYP1B1	GO:0044255	cellular lipid metabolic process
CYP2C19	GO:0044255	cellular lipid metabolic process
CYP3A5	GO:0044270	cellular nitrogen compound catabolic process
CYP1B1	GO:0044271	cellular nitrogen compound biosynthetic process
AKR1B10	GO:0044281	small molecule metabolic process
CYP1B1	GO:0044281	small molecule metabolic process
CYP2C19	GO:0044281	small molecule metabolic process
CYP3A5	GO:0044281	small molecule metabolic process
AKR1B10	GO:0044282	small molecule catabolic process
CYP1B1	GO:0045765	regulation of angiogenesis
CYP1B1	GO:0045766	positive regulation of angiogenesis
HSD17B13	GO:0045834	positive regulation of lipid metabolic process
AKR1B10	GO:0046164	alcohol catabolic process
CYP1B1	GO:0046209	nitric oxide metabolic process
AKR1B10	GO:0046247	terpene catabolic process
CYP1B1	GO:0046425	regulation of JAK-STAT cascade
CYP1B1	GO:0046427	positive regulation of JAK-STAT cascade
CYP1B1	GO:0046466	membrane lipid catabolic process
CYP2C19	GO:0046483	heterocycle metabolic process
HSD17B13	GO:0046889	positive regulation of lipid biosynthetic process
HSD17B13	GO:0046890	regulation of lipid biosynthetic process
CYP1B1	GO:0048514	blood vessel morphogenesis
CYP1B1	GO:0048518	positive regulation of biological process
HSD17B13	GO:0048518	positive regulation of biological process
CYP1B1	GO:0048519	negative regulation of biological process
CYP1B1	GO:0048522	positive regulation of cellular process
CYP1B1	GO:0048523	negative regulation of cellular process
CYP1B1	GO:0048583	regulation of response to stimulus
CYP1B1	GO:0048584	positive regulation of response to stimulus
CYP1B1	GO:0048646	anatomical structure formation involved in morphogenesis
CYP1B1	GO:0048870	cell motility
AKR1B10	GO:0050789	regulation of biological process
CYP1B1	GO:0050789	regulation of biological process
HSD17B13	GO:0050789	regulation of biological process
CYP1B1	GO:0050793	regulation of developmental process
AKR1B10	GO:0050794	regulation of cellular process
CYP1B1	GO:0050794	regulation of cellular process
CYP1B1	GO:0050877	neurological system process
AKR1B10	GO:0050896	response to stimulus
CYP1B1	GO:0050896	response to stimulus
CYP1B1	GO:0050953	sensory perception of light stimulus

CYP1B1	GO:0051090	regulation of sequence-specific DNA binding transcription factor activity
CYP1B1	GO:0051094	positive regulation of developmental process
CYP1B1	GO:0051171	regulation of nitrogen compound metabolic process
CYP1B1	GO:0051239	regulation of multicellular organismal process
CYP1B1	GO:0051240	positive regulation of multicellular organismal process
CYP1B1	GO:0051252	regulation of RNA metabolic process
CYP1B1	GO:0051270	regulation of cellular component movement
CYP1B1	GO:0051271	negative regulation of cellular component movement
AKR1B10	GO:0051606	detection of stimulus
AKR1B10	GO:0051716	cellular response to stimulus
CYP1B1	GO:0051716	cellular response to stimulus
AKR1B10	GO:0051761	sesquiterpene metabolic process
AKR1B10	GO:0051763	sesquiterpene catabolic process
CYP1B1	GO:0055114	oxidation-reduction process
CYP2C19	GO:0055114	oxidation-reduction process
CYP3A5	GO:0055114	oxidation-reduction process
CYP1B1	GO:0060255	regulation of macromolecule metabolic process
CYP1B1	GO:0061299	retina vasculature morphogenesis in camera-type eye
CYP1B1	GO:0061304	retinal blood vessel morphogenesis
AKR1B10	GO:0065007	biological regulation
CYP1B1	GO:0065007	biological regulation
HSD17B13	GO:0065007	biological regulation
CYP1B1	GO:0065008	regulation of biological quality
CYP1B1	GO:0065009	regulation of molecular function
CYP1B1	GO:0070301	cellular response to hydrogen peroxide
CYP1B1	GO:0070887	cellular response to chemical stimulus
CYP3A5	GO:0070988	demethylation
CYP3A5	GO:0070989	oxidative demethylation
CYP1B1	GO:0071310	cellular response to organic substance
CYP1B1	GO:0071407	cellular response to organic cyclic compound
CYP1B1	GO:0071603	endothelial cell-cell adhesion
CYP1B1	GO:0071840	cellular component organization or biogenesis
CYP1B1	GO:0071841	cellular component organization or biogenesis at cellular level
CYP1B1	GO:0071842	cellular component organization at cellular level
CYP1B1	GO:0080090	regulation of primary metabolic process
HSD17B13	GO:0080090	regulation of primary metabolic process
CYP1B1	GO:0090136	epithelial cell-cell adhesion
CYP1B1	GO:2000026	regulation of multicellular organismal development
CYP1B1	GO:2000112	regulation of cellular macromolecule biosynthetic process
CYP1B1	GO:2000145	regulation of cell motility
CYP1B1	GO:2000146	negative regulation of cell motility
CYP1B1	GO:2000377	regulation of reactive oxygen species metabolic process

molecular function		
AKR1B10	GO:0001758	retinal dehydrogenase activity
AKR1B10	GO:0003824	catalytic activity
CYP1B1	GO:0003824	catalytic activity
CYP2C19	GO:0003824	catalytic activity
CYP3A5	GO:0003824	catalytic activity
HSD17B13	GO:0003824	catalytic activity
AKR1B10	GO:0004033	aldo-keto reductase (NADP) activity
CYP1B1	GO:0004497	monooxygenase activity
CYP2C19	GO:0004497	monooxygenase activity
CYP3A5	GO:0004497	monooxygenase activity
CYP1B1	GO:0005488	binding
CYP2C19	GO:0005488	binding
CYP3A5	GO:0005488	binding
CYP1B1	GO:0005506	iron ion binding
CYP2C19	GO:0005506	iron ion binding
CYP3A5	GO:0005506	iron ion binding
CYP2C19	GO:0005515	protein binding
CYP2C19	GO:0008391	arachidonic acid monooxygenase activity
CYP2C19	GO:0008392	arachidonic acid epoxygenase activity
CYP2C19	GO:0008395	steroid hydroxylase activity
AKR1B10	GO:0016491	oxidoreductase activity
CYP1B1	GO:0016491	oxidoreductase activity
CYP2C19	GO:0016491	oxidoreductase activity
CYP3A5	GO:0016491	oxidoreductase activity
HSD17B13	GO:0016491	oxidoreductase activity
AKR1B10	GO:0016614	oxidoreductase activity, acting on CH-OH group of donors
AKR1B10	GO:0016616	oxidoreductase activity, acting on the CH-OH group of donors, NAD or NADP as acceptor
AKR1B10	GO:0016620	oxidoreductase activity, acting on the aldehyde or oxo group of donors, NAD or NADP as acceptor
CYP1B1	GO:0016705	oxidoreductase activity, acting on paired donors, with incorporation or reduction of molecular oxygen
CYP2C19	GO:0016705	oxidoreductase activity, acting on paired donors, with incorporation or reduction of molecular oxygen
CYP3A5	GO:0016705	oxidoreductase activity, acting on paired donors, with incorporation or reduction of molecular oxygen
CYP2C19	GO:0016709	oxidoreductase activity, acting on paired donors, with incorporation or reduction of molecular oxygen, NADH or NADPH as one donor, and incorporation of one atom of oxygen
CYP1B1	GO:0016712	oxidoreductase activity, acting on paired donors, with incorporation or reduction of molecular oxygen, reduced flavin or flavoprotein as one donor, and incorporation of one atom of oxygen
CYP2C19	GO:0016712	oxidoreductase activity, acting on paired donors, with incorporation or reduction of molecular oxygen, reduced flavin or flavoprotein as one donor, and incorporation of one atom of oxygen
CYP3A5	GO:0016712	oxidoreductase activity, acting on paired donors, with incorporation or reduction of molecular oxygen, reduced flavin or flavoprotein as one donor, and incorporation of one atom of oxygen
AKR1B10	GO:0016903	oxidoreductase activity, acting on the aldehyde or oxo group of donors
CYP2C19	GO:0018675	(S)-limonene 6-monooxygenase activity
CYP2C19	GO:0018676	(S)-limonene 7-monooxygenase activity
CYP2C19	GO:0019113	limonene monooxygenase activity

CYP1B1	GO:0019825	oxygen binding
CYP2C19	GO:0019825	oxygen binding
CYP3A5	GO:0019825	oxygen binding
CYP2C19	GO:0019899	enzyme binding
CYP1B1	GO:0020037	heme binding
CYP2C19	GO:0020037	heme binding
CYP3A5	GO:0020037	heme binding
CYP1B1	GO:0043167	ion binding
CYP2C19	GO:0043167	ion binding
CYP3A5	GO:0043167	ion binding
CYP1B1	GO:0043169	cation binding
CYP2C19	GO:0043169	cation binding
CYP3A5	GO:0043169	cation binding
AKR1B10	GO:0045550	geranylgeranyl reductase activity
CYP1B1	GO:0046872	metal ion binding
CYP2C19	GO:0046872	metal ion binding
CYP3A5	GO:0046872	metal ion binding
CYP1B1	GO:0046906	tetrapyrrole binding
CYP2C19	GO:0046906	tetrapyrrole binding
CYP3A5	GO:0046906	tetrapyrrole binding
CYP1B1	GO:0046914	transition metal ion binding
CYP2C19	GO:0046914	transition metal ion binding
CYP3A5	GO:0046914	transition metal ion binding
AKR1B10	GO:0047718	indanol dehydrogenase activity
CYP2C19	GO:0052741	(R)-limonene 6-monooxygenase activity
CYP1B1	GO:0070330	aromatase activity
CYP3A5	GO:0070330	aromatase activity
**cellular component**		
AKR1B10	GO:0000323	lytic vacuole
HSD17B13	GO:0005576	extracellular region
CYP2C19	GO:0005737	cytoplasm
CYP1B1	GO:0005739	mitochondrion
AKR1B10	GO:0005764	lysosome
AKR1B10	GO:0005773	vacuole
CYP1B1	GO:0005789	endoplasmic reticulum membrane
CYP2C19	GO:0005789	endoplasmic reticulum membrane
CYP3A5	GO:0005789	endoplasmic reticulum membrane
HSD17B13	GO:0005811	lipid particle
AKR1B10	GO:0005829	cytosol
CYP1B1	GO:0016020	membrane
CYP2C19	GO:0016020	membrane
CYP3A5	GO:0016020	membrane

CYP1B1	GO:0031090	organelle membrane
CYP2C19	GO:0031090	organelle membrane
CYP3A5	GO:0031090	organelle membrane
AKR1B10	GO:0031982	vesicle
AKR1B10	GO:0031988	membrane-bounded vesicle
AKR1B10	GO:0043226	organelle
CYP1B1	GO:0043226	organelle
CYP2C19	GO:0043226	organelle
CYP3A5	GO:0043226	organelle
AKR1B10	GO:0043227	membrane-bounded organelle
CYP1B1	GO:0043227	membrane-bounded organelle
CYP2C19	GO:0043227	membrane-bounded organelle
CYP3A5	GO:0043227	membrane-bounded organelle
AKR1B10	GO:0043229	intracellular organelle
CYP1B1	GO:0043229	intracellular organelle
CYP2C19	GO:0043229	intracellular organelle
CYP3A5	GO:0043229	intracellular organelle
AKR1B10	GO:0043230	extracellular organelle
AKR1B10	GO:0043231	intracellular membrane-bounded organelle
CYP1B1	GO:0043231	intracellular membrane-bounded organelle
CYP2C19	GO:0043231	intracellular membrane-bounded organelle
CYP3A5	GO:0043231	intracellular membrane-bounded organelle
AKR1B10	GO:0044421	extracellular region part
CYP1B1	GO:0044422	organelle part
CYP2C19	GO:0044422	organelle part
CYP3A5	GO:0044422	organelle part
AKR1B10	GO:0044424	intracellular part
CYP1B1	GO:0044424	intracellular part
CYP2C19	GO:0044424	intracellular part
CYP3A5	GO:0044424	intracellular part
HSD17B13	GO:0044424	intracellular part
CYP1B1	GO:0044425	membrane part
CYP2C19	GO:0044425	membrane part
CYP3A5	GO:0044425	membrane part
CYP1B1	GO:0044432	endoplasmic reticulum part
CYP2C19	GO:0044432	endoplasmic reticulum part
CYP3A5	GO:0044432	endoplasmic reticulum part
AKR1B10	GO:0044444	cytoplasmic part
CYP1B1	GO:0044444	cytoplasmic part
CYP2C19	GO:0044444	cytoplasmic part
CYP3A5	GO:0044444	cytoplasmic part
HSD17B13	GO:0044444	cytoplasmic part

CYP1B1	GO:0044446	intracellular organelle part
CYP2C19	GO:0044446	intracellular organelle part
CYP3A5	GO:0044446	intracellular organelle part
AKR1B10	GO:0044464	cell part
CYP1B1	GO:0044464	cell part
CYP2C19	GO:0044464	cell part
CYP3A5	GO:0044464	cell part
HSD17B13	GO:0044464	cell part
AKR1B10	GO:0065010	extracellular membrane-bounded organelle
AKR1B10	GO:0070062	extracellular vesicular exosome
